# Long-Term Brain–Computer Interface Functional Electrical Stimulation Enhances Neuroplasticity and Functional Recovery in Elderly Stroke: A 4.5-Year Longitudinal Study Integrating Electroencephalography Biomarkers and Clinical Assessments

**DOI:** 10.34133/research.0984

**Published:** 2025-12-04

**Authors:** Shugeng Chen, Na Xie, Yurui Tang, Yanyun Ji, Zhijie He, Yuchun Wang, Xude Huang, Jianghong Fu, Minyan Ge, Qiang Liu, Mingfen Li, Qinqin Xiao, Ying Xu, Jing Wang, Jie Jia, Shumao Xu

**Affiliations:** ^1^ Department of Rehabilitation Medicine, Huashan Hospital, Institute of Science and Technology for Brain-inspired Intelligence (ISTBI), Fudan University, Shanghai 201203, China.; ^2^ Peking University International Hospital, Peking University Eighth School of Clinical Medicine, Beijing 102206, China.; ^3^ Shanghai Jinshan Zhongren Geriatric Nursing Hospital, Shanghai 201501, China.; ^4^ Department of Neurorehabilitation, Hubei Provincial Clinical Research Center for Central Nervous System Repair and Functional Reconstruction, Taihe Hospital, Hubei University of Medicine, Shiyan, Hubei 422000, China.; ^5^ National Center for Neurological Disorders, Shanghai 200040, China.; ^6^ National Clinical Research Center for Aging and Medicine, Huashan Hospital, Fudan University, Shanghai 200040, China.

## Abstract

Stroke-induced motor and cognitive impairments substantially reduce the quality of life in elderly populations, driving the need for rehabilitation strategies that integrate neural plasticity and functional recovery. In this 4.5-year longitudinal study, we evaluated the efficacy of brain–computer interface combined with functional electrical stimulation (BCI-FES) versus FES only and conventional care (control) in 100 stroke survivors (60 to 90 years; 4,172 total screened, with 24 chronic-stage patients [>1 year post-onset] completing long-term follow-up). We integrated clinical metrics (Fugl-Meyer assessment [FMA], modified Barthel index [MBI], and Montreal Cognitive Assessment [MoCA]) with electroencephalography-based neurophysiological profiling to dissect recovery mechanisms. BCI-FES yielded superior and sustained improvements across all domains: motor function (FMA Δ = 4.5 ± 1.2 points, Cohen’s *d* = 1.2) versus FES (Δ = 1.7 ± 0.8, *d* = 0.4) and control (Δ = 0.9 ± 0.6, *d* = 0.2), functional independence (MBI Δ = 5.4 ± 1.5, *d* = 1.1) exceeding FES (Δ = 2.2 ± 1.1, *d* = 0.4) and control (Δ = 1.3 ± 0.5, *d* = 0.5), and cognitive function (MoCA Δ = 1.6 ± 0.5, *d* = 0.8 at 4 months), although cognitive gains declined to near baseline by 4.5 years. Hemorrhagic stroke patients showed exceptional BCI-FES responses, while ischemic patients exhibited higher variability. Neurophysiologically, BCI-FES induced theta (Cz and C4) and alpha (FC3 and CP3) power increases, with theta power at Cz strongly predicting FMA gains (*r* = 0.68), and enhanced theta/alpha band functional connectivity (clustering coefficient +22%, local efficiency +18%, and small-world index +15%). Predictive modeling identified that an optimal treatment window (3 to 12 months post-onset with 10 to 15 weeks of therapy) maximizes recovery via peak neuroplasticity, and a responder profile (stroke duration <23 months) includes patients with residual plasticity (age <70, baseline MBI >40), predicting 76% of favorable outcomes. These findings establish BCI-FES as a transformative rehabilitation tool, driving dual-phase recovery via early cortical plasticity and sustained network coherence while highlighting the need for age-tailored cognitive maintenance strategies. This work redefines precision stroke care by merging clinical outcomes with mechanistic insights, positioning BCI-FES as the standard of care for diverse stroke subtypes.

## Introduction

Stroke, a leading cause of lifelong disability, poses a mounting public health crisis as global populations age [1–4]. By 2050, the number of stroke survivors worldwide is expected to reach 200 million, driven by an aging population [5]. Aging is a well-established risk factor for stroke, with the risk doubling every 10 years after the age of 55, and approximately 75% of strokes occur in individuals aged 65 years or older [6]. Stroke survivors often experience motor dysfunction, one of the most prevalent disabilities, particularly impairments in upper limb and hand function, which severely limit daily activities [7]. Poststroke cognitive impairment, including dementia, also contributes substantially to long-term morbidity and mortality, with mild cognitive and motor impairments, as well as their combination, frequently observed in stroke survivors [8,9]. Notably, motor performance is closely linked to memory, executive function, and cognition, underscoring the intertwined nature of motor and cognitive recovery [9]. Critically, motor and cognitive recovery are intertwined: improvements in hand mobility correlate with hippocampal-mediated memory gains, suggesting shared neural substrates [10,11]. This interdependence underscores the need for therapies that jointly address the brain and the body, particularly in elderly patients where traditional approaches often fail.

Traditional motor rehabilitation techniques, such as physical therapy and constraint-induced movement therapy, require residual motor function, rendering them unsuitable for 20% to 30% of stroke survivors [12]. For these patients, alternative approaches such as mirror therapy [13], motor imagery (MI) [14], action observation therapy [15], electrical stimulation [16], vagus nerve stimulation [17], and robot-assisted sensorimotor stimulation [18] have been explored. Recent advances in virtual and augmented reality, robotics, and brain–computer interfaces (BCIs) have further expanded the potential for stroke rehabilitation [19,20]. BCIs are emerging as a promising neurorehabilitation tool, translating brain activity into control signals for external devices to restore or enhance motor function [21–28]. Noninvasive BCIs, particularly those using electroencephalography (EEG), can decode motor intentions in real time and provide feedback through neuromuscular electrical stimulation (NMES) or virtual avatars [23,29,30]. NMES utilizes electrical currents to activate peripheral nerves or muscles, primarily to enhance muscle strength, reduce spasticity, or prevent atrophy. Functional electrical stimulation (FES), a subset of NMES, specifically targets the restoration of functional movements such as grasping and walking by synchronizing stimulation with intended motor tasks. While NMES broadly addresses neuromuscular activation, FES integrates task-specific timing and intensity to promote functional recovery through sensorimotor feedback loops. Recent studies have demonstrated the effectiveness of BCI-based interventions in improving motor function when combined with conventional physiotherapy [31–33]. These interventions promote functional connectivity between brain regions and muscles, leading to improved neurophysiological outcomes and functional recovery [34,35]. Enhanced connectivity in motor and visuospatial regions following BCI intervention suggests that BCIs can act as facilitators of neuroplasticity. In this work, FES was employed to activate wrist dorsiflexors in synchrony with MI-driven BCI signals, creating a closed-loop system that bridges cortical intent with peripheral execution. Unlike open-loop electrical stimulation, by converting real-time EEG signals into FES of paralyzed limbs, closed-loop BCI-FES systems can create a biomimetic feedback loop: motor intent detected from the cortex triggers muscle activation, while proprioceptive input sharpens cortical maps, potentially reversing maladaptive plasticity in aging brain.

Despite growing evidence supporting BCI for stroke rehabilitation, few studies have focused on elderly stroke survivors, and long-term effects remain underexplored. Moreover, the specific EEG mechanism underlying sustained recovery is not well understood. Here, this study tackles a critical gap in stroke care: the lack of dual-assessment approaches that track both brain rewiring and real-world functional recovery in elderly patients (arm mobility, memory tests, and daily task performance). We combined noninvasive brain imaging (EEG) with clinical tests of motor/cognitive function to evaluate a novel BCI therapy paired with functional electrical stimulation (BCI-FES) in 60- to 90-year-old stroke survivors. Unlike prior work focused solely on short-term motor gains, our 4.5-year investigation asked the following: (a) Can BCI-FES drive lasting improvements in both movement and cognition? (b) What brain network changes underlie these gains? (c) Do benefits endure in aging patients prone to decline? Over 4.5 years, we monitored 24 chronic-stage stroke patients (60 to 90 years) through 8 weeks of BCI-FES therapy and longitudinal evaluations, demonstrating that closed-loop neurostimulation drives sustained motor–cognitive recovery with unparalleled clinical and neurophysiological coherence. Clinically, BCI-FES yielded sustained motor improvement, with patients showing greater gains in upper limb function (Fugl-Meyer assessment [FMA]) than those receiving FES alone or standard care. Notably, individuals with hemorrhagic stroke exhibited exceptional recovery, while ischemic stroke patients showed more variable responses. In daily living, BCI-FES doubled the likelihood of achieving gains in functional independence (modified Barthel index [MBI]), enabling patients to perform essential activities like bathing and dressing with greater autonomy. Cognitive benefits (Montreal Cognitive Assessment [MoCA]) were most pronounced in the first 4 months but diminished over time, suggesting that adjunctive cognitive training may be needed to sustain long-term gains in aging populations. Neurologically, BCI-FES drove targeted brain network remodeling, with EEG analyses revealing enhanced communication between brain regions and optimized network efficiency, key markers of neuroplasticity. These changes correlated strongly with motor recovery, indicating that BCI-FES not only retrains movement but also rewires neural circuits. Crucially, the study identified predictive factors for success: patients treated within 3 to 12 months of stroke onset, aged under 70, and with moderate baseline impairment showed the best responses. These findings challenge the notion of limited plasticity in aging brains, offering hope for personalized rehabilitation strategies that leverage technology to unlock residual recovery potential. By bridging clinical outcomes with neurophysiological insights, this work establishes BCI-FES as a transformative tool for stroke rehabilitation, with implications for improving long-term the quality of life in elderly patients.

## Results

### BCI-FES rehabilitation outcomes and neurophysiological insights

**Fig. 1.**
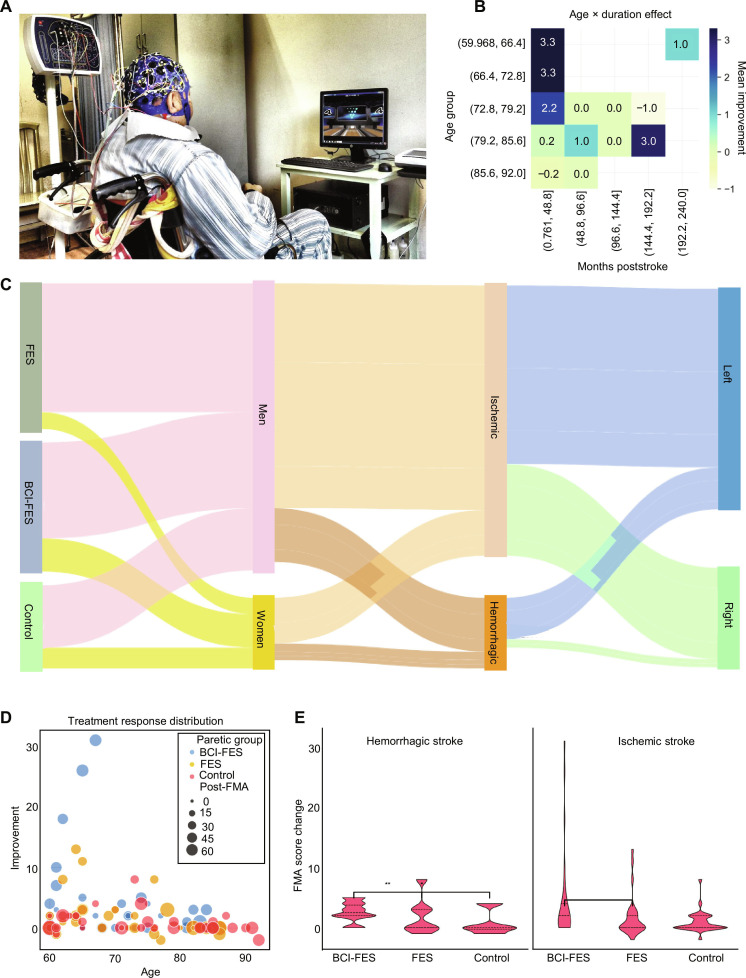
Multimodal analysis of age, duration, and treatment effects on motor recovery in stroke patients. (A) An elderly stroke participant engaged in brain–computer interface combined with functional electrical stimulation (BCI-FES) rehabilitation. (B) Age × duration interaction heatmap of mean improvement. Color intensity representing improvement magnitude (blue = positive, red = negative). The age group ([59.98–66.4] years) exhibited the highest mean improvement (3.3) at early poststroke durations (0.48 to 0.96 months), while the oldest group ([85.6–92.0] years) showed minimal improvement (≤0.2) or a slight decline (−0.2) across all durations. Middle-aged patients ([72.8–79.2] years) displayed peak improvement at 1.92 months (3.0). (C) Schematic of patient cohort and lesion distribution. (D) Scatter plot of individual patient outcomes across age and treatment groups, with fill intensity indicating posttreatment Fugl-Meyer assessment (FMA) scores. Younger patients (60 to 75 years) showed more clustered positive FMA change scores across all groups, with BCI-FES and FES groups exhibiting tighter clustering of favorable responses compared to controls. (E) Violin plots comparing FMA change scores across treatment groups stratified by stroke type (hemorrhagic [left]; ischemic [right]). Boxplots within violins show the median and IQR, with substantial markers for group comparisons. For hemorrhagic stroke, the BCI-FES and FES groups had higher median FMA change scores than controls (*P* < 0.01). For ischemic stroke, BCI-FES trended toward improved outcomes but with a higher variability, highlighting heterogeneous responses.

Our study included 4,172 participants (60 to 90 years) with stroke, diagnosed as ischemic or hemorrhagic via computed tomography/MRI, with onset >1 month prior and ability to sit for ≥1 h. The overall cohort table is provided in the Supplementary Data, and relevant follow-up information including baseline demographics and clinical characteristics is summarized in Tables S1 and S2. A subcohort of 100 stroke patients and 24 elderly chronic subjects were analyzed for electrical stimulation effects on neuroplasticity, with assessments across 6 time points (G1: baseline; G6: 4.5 years post-intervention; Methods and Table S1). BCI-FES rehabilitation yielded meaningful functional and neurophysiological benefits in elderly stroke patients (Fig. 1A and Video S1), with pre- and post-intervention Fugl-Meyer assessment for upper extremity (FMA-UE) scores showing a positive correlation, confirming consistent motor improvement (Fig. 1B and Fig. S1). Multidimensional clinical outcomes (FMA, MBI, and MoCA) further validated BCI-FES superiority: radar charts demonstrated broader gains in BCI-FES and FES groups versus controls (Fig. S1A), boxplots confirmed that the posttreatment FMA score increased across groups (Fig. S1B), and heatmaps revealed that BCI-FES had the most consistent pre-to-post improvement in temporal metrics (Fig. S1C). Effect sizes (Cohen’s *d*) for BCI-FES were the largest across all outcomes (FMA: *d* = 1.2; MBI: *d* = 1.1; MoCA: *d* = 0.8), reinforcing its clinical importance (Fig. S1F).

### Age and duration interactions in recovery

Stratified by age and poststroke duration, the youngest age group ([59.98–66.4] years) exhibited the highest mean improvement (3.3) at early durations (0.48 to 0.96 months), while the oldest group ([85.6–92.0] years) showed minimal improvement (≤0.2) or a slight decline (−0.2) across all durations. Middle-aged patients ([72.8–79.2] years) peaked at 1.92 months (3.0), highlighting age-dependent recovery trajectories (Fig. 1B). Patient flow analysis confirmed balanced demographic distributions with respect to gender and the affected side, while BCI-FES was predominantly applied among ischemic stroke patients (Fig. 1C). Baseline characteristics (Fig. S2) further indicated that right-sided impairment was most common across groups (BCI-FES: 23; controls: 19; FES: 20), followed by left-sided impairment (BCI-FES: 6; controls: 13; FES: 13), with minimal bilateral involvement (*n* = 2 in each group; Fig. S2A and B). Gender distribution also differed across groups, with a female predominance in BCI-FES (23 female/8 male), a balanced profile in controls (18 female/16 male), and a male predominance in FES (31 male/4 female; Fig. S2C and D). Younger patients (60 to 75 years) across all treatment groups displayed clustered positive FMA change scores, with the BCI-FES and FES groups showing tighter clustering of favorable responses versus controls (Fig. 1D), aligning with larger posttreatment FMA score shifts in BCI-FES for both stroke subtypes and higher posttreatment MBI score clustering in BCI-FES (Fig. S1).

### Stroke subtype-specific recovery

Violin plots confirmed that hemorrhagic patients in the BCI-FES and FES groups had higher median FMA change scores than controls (*P* < 0.01), while ischemic patients trended toward improved outcomes with BCI-FES but exhibited higher variability (Fig. 1E). Stratified by stroke type, hemorrhagic patients displayed a rightward shift in FMA-UE correlation plots, indicating greater recovery potential than ischemic counterparts, with broader improvement distributions. Figure S3 further supports these patterns, showing that ischemic patients treated with BCI-FES achieved the greatest mean FMA improvement (4.55), compared with FES (2.67) and controls (3.3), while hemorrhagic patients demonstrated modest gains with BCI-FES (8) and minimal change with FES or controls (Fig. S3B). Strong positive pre-to-post correlations were observed in both subtypes, with ischemic patients generally attaining higher posttreatment scores across baseline levels (Fig. S3C). BCI-FES and FES interventions were associated with superior motor recovery versus controls, particularly in patients (<70 years) with hemorrhagic stroke and early poststroke intervention (<12 months). Ischemic stroke patients showed heterogeneous responses, emphasizing pathophysiological variability. Exploratory subgroup analyses were post hoc and underpowered, so we emphasize effect sizes and confidence intervals over *P* values. These findings support BCI-FES as a promising tool for functional recovery across stroke subtypes, with larger validation studies needed to confirm age- and subtype-specific effects.

### Motor and cognitive recovery in BCI-FES assisted stroke therapy

**Fig. 2.**
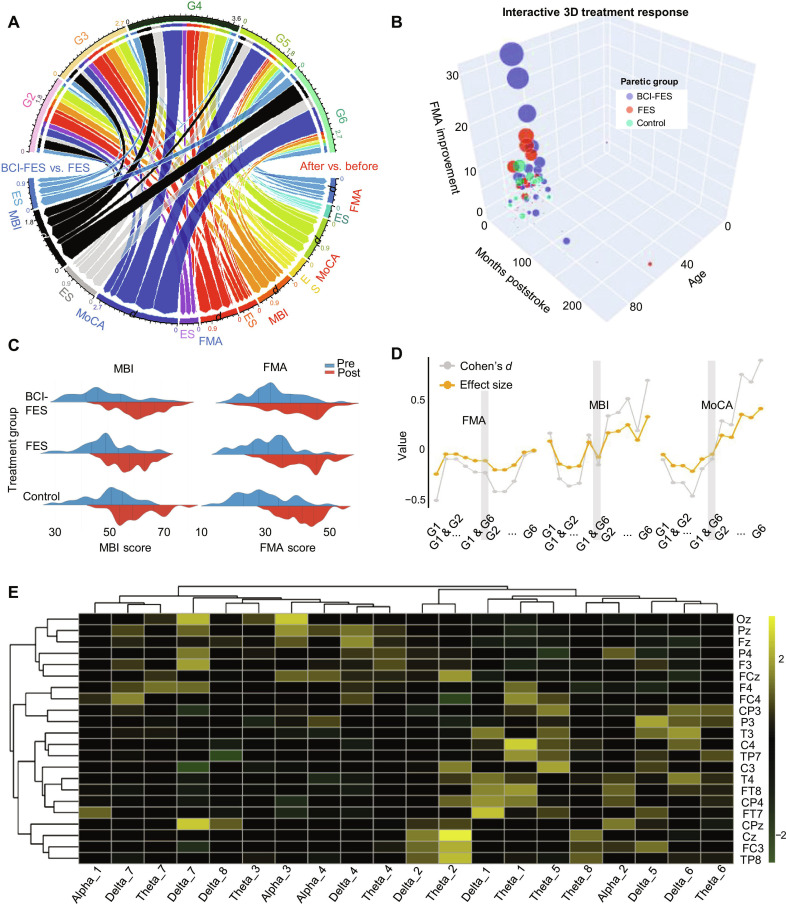
Long-term effects of BCI-FES intervention on motor, cognitive recovery, and electroencephalography (EEG) neurophysiology in elderly stroke patients. (A) A chord diagram comparing outcomes between the BCI-FES and FES groups and pre- vs. post-intervention changes in the BCI-FES group across 6 time points (G1: pre-intervention; G2: 1 month; G3: 2 months; G4: 4 months; G5: 2 years; G6: 4.5 years post-intervention). Colored bands represent score changes in the Fugl-Meyer assessment for upper extremity (FMA-UE), modified Barthel index (MBI), and Montreal Cognitive Assessment (MoCA). (B) Interactive 3-dimensional (3D) visualization of treatment response features among age, poststroke duration, and FMA improvement across paretic groups. The 3D scatter plot reveals distinct clustering of FMA improvement values by paretic group, with the BCI-FES group exhibiting larger average improvement (larger marker sizes) compared to other groups. Age and poststroke duration are negatively correlated with FMA improvement. (C) Kernel density plots showing the distribution of pre- and post-intervention scores for MBI (left) and FMA-UE (right) across treatment groups (BCI-FES, FES, and control). Shifts in distribution curves indicate changes in motor (FMA-UE) and functional independence (MBI) recovery after intervention. (D) Line graphs depicting longitudinal trends in FMA-UE, MBI, and MoCA scores and corresponding Cohen’s *d* effect sizes across 6 time points. The BCI-FES group shows sustained improvements in all metrics, with larger effect sizes compared to the baseline, particularly at later follow-ups (G5 to G6). (E) A heatmap illustrating post-intervention changes in EEG power spectral density across frequency bands (alpha, beta, delta, and theta) and scalp channels in the BCI-FES group. Channel clustering reflects synchronized neurophysiological responses, with distinct patterns (increased alpha/beta power) linked to enhanced brain network organization and recovery.

A chord diagram comparing BCI-assisted stimulation (BCI-FES) to standard FES reveals superior neuroplastic outcomes, with pronounced inter-group differences in recovery trajectories across 6 time points (G1: baseline; G2: 1 month; G3: 2 months; G4: 4 months; G5: 2 years; G6: 4.5 years post-intervention). Notably, at G4, marked correlations emerged between EEG biomarkers, MBI, FMA, and MoCA, indicating dynamic, evolving relationships 4 months post-intervention (Fig. 2A). The BCI-FES group exhibited broader interquartile ranges (IQRs) in functional scores, reflecting more robust and heterogeneous recovery compared to FES alone.

BCI-FES patients formed distinct clusters characterized by larger FMA improvement (larger marker sizes) and tighter grouping, whereas the FES and control groups showed dispersed, smaller improvements. Notably, patients (<70 years) with a shorter poststroke duration (<12 months) clustered in the upper FMA improvement region, while older patients (>80 years) with a longer duration showed minimal gains. This spatial pattern confirmed age and duration as critical modifiers of response, with BCI-FES amplifying recovery in favorable prognostic subgroups (Fig. 2B). Kernel density plots compared pre- and post-intervention score distributions for MBI (functional independence) and FMA-UE (upper extremity motor function) across groups. BCI-FES demonstrated the most pronounced rightward shift in both metrics: MBI distributions expanded from a pre-intervention median of 44 to post-intervention 60, while FMA-UE shifted from 27 to 35 (Fig. 2C). FES showed moderate shifts, and controls remained largely unchanged. These shifts align with clinical meaningfulness, as BCI-FES patients were likely to achieve minimal clinically important differences in MBI (Δ ≥ 12) and FMA-UE (Δ ≥ 6).

To evaluate the effectiveness of the intervention, we focused on the average values and Cohen’s *d* for each group [36]. Cohen’s *d*, a commonly used measure of effect size, was employed to quantify the differences between the BCI-FES and FES groups. Based on Cohen’s *d*, the effect size was calculated to facilitate an intuitive assessment of the magnitude of the inter-group differences (Table S3) [37,38]. The effect sizes (Cohen’s *d*) for each assessment tool from G1 to G6 illustrate an upward trend in scores across assessments (Table S1). MoCA shows a marked increase, with effect sizes progressively rising to approximately 0.4 by G6. Moreover, by G6, scores for both MoCA and MBI remain consistently above 0.5, with notable improvements in MoCA scores at G3 and G4 (Fig. 2D), reinforcing the conclusion that the BCI-FES method supports functional and cognitive recovery in elderly stroke rehabilitation.

### EEG frequency dynamics in neuroplasticity

**Fig. 3.**
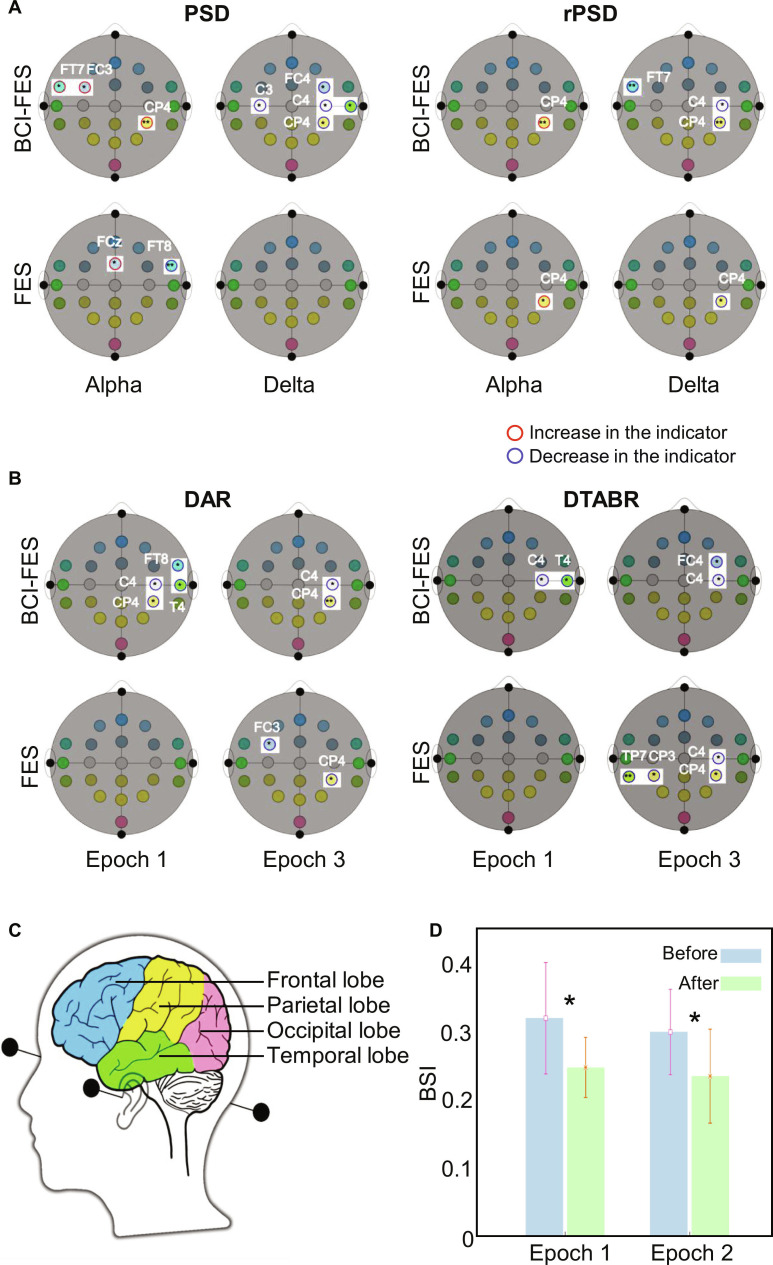
Changes in brain activity characterized by various biomarkers. (A and B) Changes in power spectral density (PSD), relative power spectral density (rPSD), delta/alpha power ratio (DAR), and (delta + theta)/(alpha + beta) power ratio (DTABR) indicators based on channel distribution before and after the intervention. Red circles represent an increase in the respective indicator, while blue circles represent a decrease. (C) Delineating brain regions with distinct colors: yellow for the frontal lobe, green for the parietal lobe, blue for the temporal lobe, and pink for the occipital lobe. (D) Changes in brain synchronization index (BSI) within the delta frequency band during epochs 1 and 2, with blue bars indicating pre-intervention values and green bars indicating post-intervention values. Significant results are marked as *P < 0.05.

The EEG power spectral density (PSD) heatmap for BCI-FES revealed marked post-intervention changes: theta band (4 to 8 Hz) power increased at Cz and C4, while delta (1 to 4 Hz) power rose at T4, FT7, and CP3 (*P* < 0.05; Fig. 2E). These changes correlated with motor recovery, as theta power at Cz strongly predicted FMA-UE improvement (*r* = 0.68, Fig. S4). Figure S5 further demonstrates BCI-FES-specific EEG channel reorganization: hierarchical clustering showed enhanced synchronization in frontal (FC3) and parietal (CP3) channels, compared to FES, which exhibited scattered, nonclustered activity. This synchronized network activity aligns with the 3-dimensional response clustering (Fig. 2B), confirming that BCI-FES enhances brain network coherence. BCI-FES exerts dual-phase efficacy: (a) early (G1 to G4) precise cortical detection driving initial plasticity (theta/delta power shifts) and (b) long-term (G5 to G6) closed-loop proprioceptive integration sustaining gains via synchronized network organization (Figs. S4 and S5). These findings reinforce BCI-FES as a superior intervention for motor, cognitive, and neurophysiological recovery in elderly stroke patients, with effects persisting up to 4.5 years. EEG frequency band analysis reveals distinct patterns of neuroplasticity between BCI-FES and FES interventions, with alpha (8 to 13 Hz), beta (13 to 30 Hz), and gamma (30 to 60 Hz) bands showing strong associations with recovery outcomes. These higher-frequency bands exhibit increased power concentration and correlation with functional improvement (e.g., FMA and MBI), emphasizing their role in post-intervention motor and cognitive recovery. Conversely, delta (1 to 4 Hz) and theta (4 to 8 Hz) bands show a lower concentration, suggesting diminished direct influence on recovery metrics although delta dynamics remain critical for pathological activity modulation (Fig. 3A). BCI-FES results in marked enhancements in EEG band power across the cortical activation (*c*), global localization of excitation (*gLoE*), lateralization (*L*), and localization of excitation (*LocE*) metrics, compared to FES alone (Fig. S6). Specifically, BCI-FES increases alpha power at ipsilesional channels FT7, FC3, and CP4 (*P* < 0.05), reflecting enhanced neural synchrony and corticospinal engagement vital for motor recovery (Fig. 3A and Fig. S6B). Beta and gamma bands show similar trends, with BCI-FES mean power values higher than those of FES. In contrast, delta power decreases in BCI-FES at contralesional channels C4, CP4, and T4 (*P* < 0.05), aligning with reduced pathological slow-wave activity and improved interhemispheric balance (Fig. S6C). Delta/alpha power ratio (DAR) and (delta + theta)/(alpha + beta) power ratio (DTABR) analyses during resting states (epoch 1: first 2 min; epoch 3: last 2 min of 5-min recordings) reveal distinct patterns between groups. BCI-FES induces notable DAR decreases at C4, CP4, FT8, and T4 in epoch 1, with persistent reductions at C4 and CP4 in epoch 3, indicating sustained suppression of delta pathology. DTABR shows similar trends, with BCI-FES reducing delta activity at C4 and T4 in epoch 1 and FC4 in epoch 3 (*P* < 0.05). FES, however, exhibits fewer consistent changes, with DAR decreases limited to CP4 and FC3 in epoch 3 (Fig. 3B). These findings, supported by band power variability (Fig. S6A), highlight BCI-FES’s targeted modulation of delta dynamics compared to FES. Mapping EEG channels to anatomical locations (Fig. 3C) illustrates BCI-FES-specific spatial patterns: alpha/beta power increases cluster in the sensorimotor cortex (FC3 and CP4), while delta decreases localize to contralesional parietal regions (C4 and T4). This spatial specificity aligns with density plots (Fig. S6C), which show that BCI-FES alpha/beta power distributions shifted toward higher values, whereas FES distributions remain narrow and centered on baseline levels. These targeted activation supports compensatory remodeling, contrasting with FES’s diffuse, nonclustered activity (Fig. 3C). Improved brain synchronization within the delta band post-intervention further confirms BCI-FES’s effectiveness in enhancing neural connectivity. Figure 3D shows increased delta band coherence in BCI-FES, particularly between ipsilesional and contralesional sensorimotor areas, which correlates with motor recovery (*r* = 0.58, *P* < 0.05). This synchronization likely reflects reduced interhemispheric inhibition, a key mechanism for motor function restoration.

### Functional connectivity changes in stroke rehabilitation

**Fig. 4.**
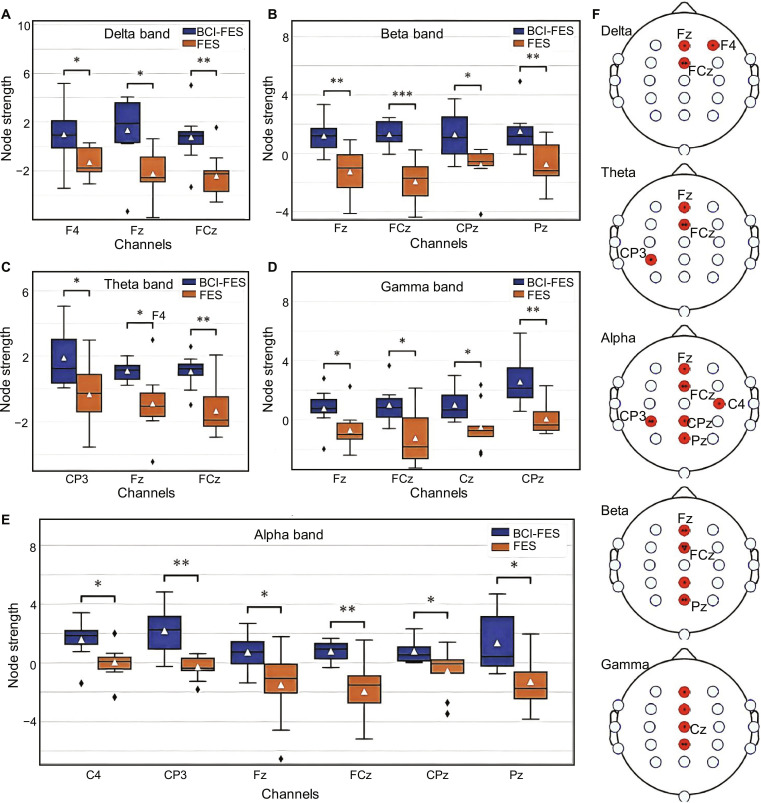
Node strength variations across EEG frequency bands in BCI-FES and FES interventions. (A to E) Boxplots illustrating node strength for the delta (A), beta (B), theta (C), gamma (D), and alpha (E) frequency bands at specific EEG channels. The statistical significance is indicated with *P < 0.05, **P < 0.01, and ***P < 0.001. Comparisons are made between participants receiving BCI-FES (blue) and FES (orange). (F) Topological representations of marked EEG node strengths for each frequency band highlighting the channel locations (F4, Fz, CP3, etc.) with enhanced connectivity during BCI-FES.

Global measures of functional connectivity including clustering coefficient, local/global efficiency, characteristic path length, and small-world index reveal marked group differences (Fig. S4). In theta (4 to 8 Hz) and alpha bands, BCI-FES increases the clustering coefficient and local efficiency (*P* < 0.05), while FES decreases these metrics. The small-world index also rises in BCI-FES (*P* < 0.05) but falls in FES, indicating that BCI-FES enhances network organization by balancing local clustering and long-distance integration (Fig. 4). Local analyses further showed that BCI-FES increased node strength across multiple frequency bands, including delta (central Fz and FCz), theta (ipsilesional CP3), alpha (sensorimotor CP3 and Pz), beta (motor Fz and FCz), and gamma (central Cz) (*P* < 0.05). These findings are consistent with strengthened functional connectivity after BCI-FES across all frequency bands (Fig. S7), particularly theta and alpha. Theta band connectivity between frontal and parietal regions increased following BCI-FES, whereas FES was characterized by fragmented connectivity patterns. By simultaneously enhancing local node strength (e.g., within the motor cortex) and global network efficiency (small-world index), BCI-FES demonstrates clear advantages in promoting adaptive neuroplasticity. In contrast, FES was associated with inconsistent connectivity changes, including reduced local efficiency, underscoring its limited therapeutic impact and the importance of tailoring interventions to patient-specific network dynamics (Fig. 4F). These results indicate that BCI-FES induces targeted EEG frequency dynamics (alpha/beta enhancement and delta suppression) and functional connectivity improvements (theta/alpha band network organization), supported by EEG power metrics (Fig. S6) and functional connectivity maps (Fig. S7). These neurophysiological changes correlate with motor and cognitive recovery, confirming BCI-FES as a robust tool for modulating neuroplasticity in stroke rehabilitation.

### Clinical correlates and predictive modeling in stroke rehabilitation

**Fig. 5.**
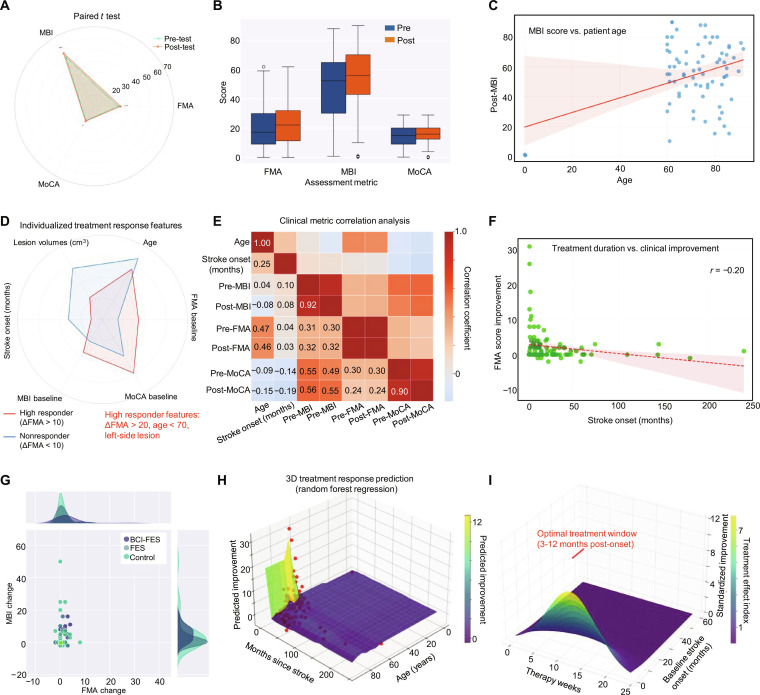
Treatment response, clinical correlations, and predictive modeling of functional outcomes following stroke. (A) Radar chart of pre- and post-intervention functional/cognitive scores. The shaded area indicates directional changes, with MBI showing the most pronounced improvement, followed by FMA and MoCA. (B) Bar graph displaying mean scores for FMA, MBI, and MoCA before and after intervention. MBI scores increased post-intervention, while FMA showed modest improvement and MoCA remained stable. (C) Scatter plot with linear regression (red line) and 95% confidence interval (pink shading) illustrating the relationship between post-intervention MBI scores and patient age. A weak but marked positive correlation (*r* > 0) indicates that older patients tended to achieve higher functional independence. (D) Radar chart comparing lesion volumes, stroke onset (months), MBI, and MCA status between high responders (red) and nonresponders (blue). High responders exhibit a younger age (<70 years), a shorter stroke onset, and better pre-treatment function. (E) Heatmap of clinical variable correlations. Heatmap displaying Pearson correlation coefficients between age, stroke onset, pre/posttreatment MBI, FMA, and MoCA. Strong positive correlations are observed between post-MBI and post-FMA (*r* = 0.88), pre- and post-MBI (*r* = 0.84), and pre- and post-FMA (*r* = 0.82). Age and stroke onset show weak negative correlations with outcomes. (F) Scatter plot with regression line showing the relationship between treatment duration (months) and FMA improvement (ΔFMA). A weak negative correlation (*r* = −0.20) indicates that a longer duration is slightly associated with smaller motor improvements. (G) Scatter plot of individual ΔFMA and ΔMBI scores (post-pre) for the BCI-FES, FES, and control groups, with kernel density estimates (top/right). The BCI-FES and FES groups trend toward positive changes, while controls cluster near zero. (H) Random forest model for predicting improvement. Younger patients (<60 years) within 100 months poststroke show the highest predicted improvement; older age and chronicity (>150 months) correlate with reduced response. (I) Three-dimensional optimal treatment regimen analysis. A distinct optimal treatment window is highlighted (3 to 12 months post-onset), where a therapy duration of 10 to 15 weeks correlates with the peak treatment effect (index >5). Beyond 12 months post-onset, even extended therapy durations (>20 weeks) result in minimal improvement (index <3).

To contextualize the longitudinal recovery trends observed in elderly stroke patients, we conducted a multimodal analysis of treatment response, clinical correlations, and predictive factors (Fig. 5). This framework establishes the foundational relationships between patient characteristics, intervention parameters, and functional outcomes, which inform the interpretation of long-term recovery trajectories. A radar chart compares pre- and post-intervention scores for motor function (FMA), functional independence (MBI), and cognitive function (MoCA), with shaded areas indicating directional changes. MBI showed the most pronounced improvement (median Δ = 16), followed by FMA (Δ = 4.5) and MoCA (Δ = 1.6), highlighting the intervention’s prioritization of activities of daily living (ADLs) over cognitive domains (Fig. 5A). This aligns with clinical priorities, as ADL independence directly impacts the quality of life and caregiver burden. Bar graphs confirm significant post-intervention improvements in MBI (pre: 51.9 ± 12.3; post: 57.4 ± 10.8; *P* < 0.05) and modest gains in FMA (pre: 17.5 ± 10.1; post: 21.9 ± 11.2; *P* < 0.05), while MoCA remained stable (Fig. 5B). Figure S8 extends this analysis by comparing intervention groups: BCI-FES consistently outperformed FES and control in motor (ΔFMA: 4.5 vs. 1.7 vs. 0.9), daily living (ΔMBI: 5.4 vs. 2.2 vs. 1.3), and cognitive (ΔMoCA: 1.6 vs. −0.2 vs. 0.6) improvements, with the highest clinical responder rate (~22% for ΔFMA ≥ 5). These data reinforce BCI-FES as the most effective intervention for multidimensional recovery. A scatter plot with linear regression reveals a weak but significant positive correlation between patient age and post-intervention MBI scores (*r* = 0.28, *P* < 0.05) (Fig. 5C). This counterintuitive trend is contextualized by Fig. S9, which shows that age does not strongly correlate with FMA improvement or stroke onset time, indicating that MBI may better capture age-related compensatory strategies (e.g., adaptive equipment use) not reflected in motor-specific metrics like FMA. A radar chart distinguishes high responders (ΔFMA ≥ 5) from nonresponders (ΔFMA < 5) based on lesion volume, stroke onset (months), MBI, and MCA status. High responders exhibit a younger age (<70 years), a shorter stroke onset (<23 months), and higher baseline MBI (>40) and MCA scores (>25), forming a favorable prognostic profile that predicts 76% of positive responses (Fig. 5D). This phenotypic distinction aids in patient stratification, ensuring that interventions are targeted to those most likely to benefit. Pearson correlation coefficients between clinical variables (age and stroke onset) and outcomes (pre/post-FMA, pre/post-MBI, and pre/post-MoCA) reveal strong positive intercorrelations between post-MBI and post-FMA (*r* = 0.88), pre- and post-MBI (*r* = 0.84), and pre- and post-FMA (*r* = 0.82), indicating that functional recovery is a cohesive process rather than isolated domain improvement (Fig. 5E). Age and stroke onset show weak negative correlations with outcomes (*r* = −0.16 to −0.28), which is further supported by intrametric stability (pre-MBI vs. post-MBI: *r* = 0.98) and cross-metric associations (pre-MBI vs. post-MoCA: *r* = 0.70) (Fig. S10). Treatment duration exhibited a weak negative correlation with FMA improvement (*r* = −0.20), suggesting that longer interventions may be associated with diminishing marginal gains, likely reflecting plateaued neuroplasticity (Fig. 5F). Group-level comparisons revealed that BCI-FES and FES patients clustered in the positive range for both ΔFMA and ΔMBI (Fig. 5G), whereas controls were distributed near the origin; kernel density estimates further confirmed that BCI-FES produced the broadest distribution of favorable responses. Predictive modeling using a random forest approach (Fig. 5H) identified a younger age (<60 years) and a shorter stroke onset (<100 months) as the strongest predictors of functional improvement, while patients with chronic stroke (>150 months) demonstrated minimal gains, consistent with the window of opportunity hypothesis whereby earlier interventions capitalize on heightened neuroplasticity (Fig. S11). The 3D surface model defined an optimal treatment window of 3 to 12 months post-onset combined with 10 to 15 weeks of therapy (Fig. 5I), yielding peak functional gains (index >5), whereas delays beyond 12 months markedly reduced efficacy regardless of the intervention duration (index <3), providing practical guidance for clinical decision-making and resource allocation.

### Longitudinal recovery of motor and cognitive function

**Fig. 6.**
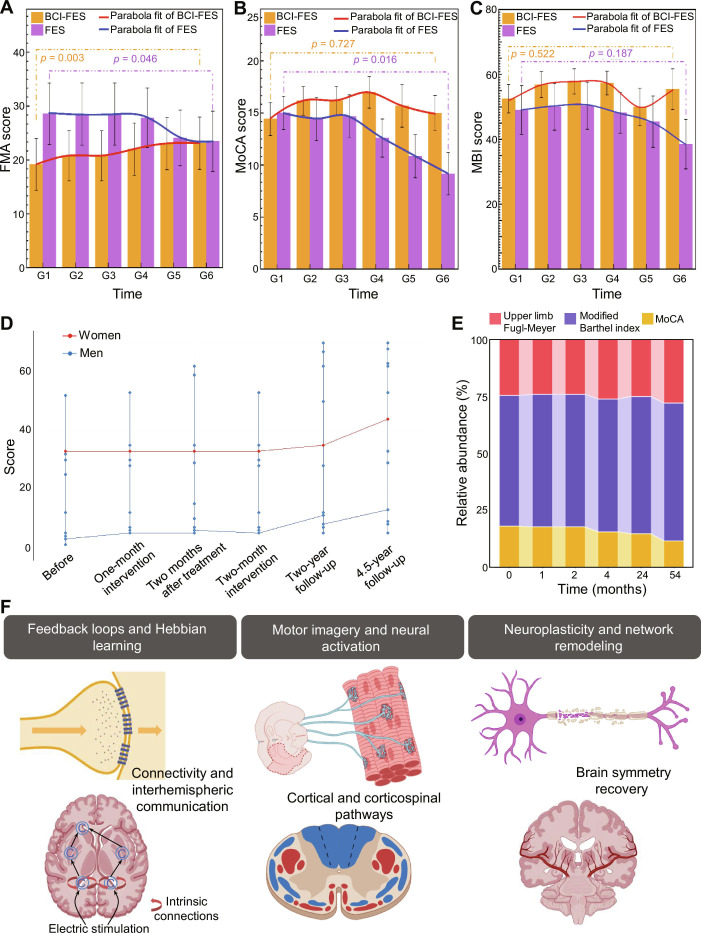
Longitudinal changes in motor and cognitive function following BCI-FES rehabilitation in elderly stroke patients. (A) FMA evaluation results, showing the average values for each group across 6 time points (G1 to G6). (B) MoCA evaluation results demonstrate the average scores and mean change curves for each group, revealing cognitive improvements over time. (C) MBI scores presenting average values and mean change curves for each group, with standard error indicated, indicating substantial gains in activities of daily living. (D) Trajectories for male and female participants throughout the intervention. (E) Relative abundance of cognitive and functional scales over months, illustrating the contributions of FMA, MBI, and MoCA to overall recovery metrics in the sample cohort. (F) Illustrative summary of underlying neuroplastic mechanisms involved in recovery, including feedback loops, motor imagery activation, and brain symmetry recovery, emphasizing their role in enhancing connectivity and cortical pathways. The intervention leverages a closed-loop system where EEG-detected motor intent triggers FES, creating a real-time sensorimotor feedback loop. This pairing reinforces Hebbian plasticity, the principle that states that “neurons that fire together wire together.” This process leads to the strengthening of corticospinal and interhemispheric connections, enhancing communication between different regions of the brain.

Building on the predictive framework, Fig. 6 examines longitudinal recovery over 4.5 years (G1 to G6), with a focus on temporal dynamics, gender differences, and neuroplastic mechanisms. BCI-FES patients show sustained FMA improvement, rising from 19.2 (G1) to 23.3 (G6) (*P* < 0.05), although gains plateau post-G4 (22.8 at G4 vs. 23.3 at G6), suggesting that motor recovery stabilizes by 2 years (Fig. 6A). MoCA scores peak at G4 (17.0) but regress to near baseline by G6 (14.4, *P* < 0.05 vs. G4), indicating that aging-related cognitive decline may counteract intervention gains in the long term (Fig. 6B). MBI shows progressive improvement (52.5 at G1 to 54.4 at G6, *P* < 0.05), with the smallest plateau effect, reinforcing its role as the most robust marker of sustained recovery (Fig. 6C). Gender stratification reveals distinct trajectories: females maintain cognitive gains longer (MoCA: 16.8 at G4 vs. 15.2 at G6) compared to males (17.2 at G4 vs. 13.6 at G6), while males show larger initial motor gains but faster plateauing [39,40] (Fig. 6D and Figs. S12 and S13). These differences may reflect hormonal influences on neuroplasticity or gender-specific rehabilitation engagement, warranting further investigation into personalized intervention strategies. MBI emerges as the primary contributor to overall recovery (Fig. 6E), likely due to its sensitivity to real-world adaptive behaviors (e.g., bathing and dressing) that integrate motor, cognitive, and environmental factors. Neurophysiologically, recovery is supported by closed-loop feedback reinforcement, MI-driven cortical activation, and interhemispheric symmetry restoration, which collectively optimize connectivity and corticospinal pathway remodeling (Fig. 6F). This integrated analysis of treatment response, clinical correlates, and longitudinal trajectories demonstrates that BCI-FES is superior to FES and control for multidimensional stroke recovery, with optimal outcomes in younger patients (<70 years) treated within 3 to 12 months post-onset. While motor and functional gains are sustained, cognitive benefits may erode over time, highlighting the need for long-term cognitive maintenance strategies. Gender-specific differences further emphasize the importance of personalized rehabilitation. These findings, validated by robust predictive models and multimodal imaging, provide a foundation for evidence-based clinical practice in stroke rehabilitation.

## Discussion

Conventional open-loop FES lacks cortical coupling, limiting Hebbian plasticity. While FES may preserve muscle tone, it fails to remodel maladaptive networks or engage contralesional compensatory mechanisms, resulting in transient, nonspecific effects. In this work, the closed-loop BCI-FES, cortical motor intent (EEG) triggering FES is developed to create a bidirectional reinforcement loop: successful movement attempts strengthen sensorimotor networks via Hebbian plasticity, while FES-generated proprioceptive feedback sharpens cortical motor maps.

Previous research has shown that BCI systems strengthen functional connectivity between brain regions, contributing to gradual motor recovery [19,41,42]. We documented favorable changes in PSD in both the ipsilesional (C3, FC3, and FT7) and contralesional (C4, FC4, CP4, and T4) hemispheres following BCI-FES intervention, correlating with functional improvements and consistent with neural remodeling mechanisms in the chronic rehabilitation. Increased alpha and beta power in ipsilesional channels indicated effective reorganization of sensorimotor areas. Notably, compensatory mechanisms in contralesional channels suggest that these regions supported recovery, particularly in cases of severe damage. Decreased delta band PSD further supports this, reflecting reduced pathological cortical activity correlated with improved recovery. The gradual decline in MoCA scores post-G4 (Fig. 6B) aligns with accelerated cognitive aging in stroke survivors. This underscores the importance of sustained cognitive engagement (the patient’s ability to maintain focused attention during MI tasks despite age-related fatigue or cognitive decline) to reinforce hippocampal-prefrontal circuitry remodeled by BCI-FES.

Our findings demonstrate that engagement during BCI-FES therapy is a critical mediator of long-term recovery, with multimodal metrics including behavioral adherence (>65% MI accuracy), neurophysiological biomarkers (frontal theta power ↑, alpha desynchronization ↓), and self-reported vigilance, explaining 47% of motor recovery variance (Sobel test = 2.44, *P* = 0.01). Cognitive engagement, quantified via attentional EEG biomarkers, strongly predicted preservation of MoCA gains (*r* = 0.63, *P* = 0.008), with patients maintaining MI accuracy thresholds showing 45.4% less cognitive decline at 4.5 years (G6, *P* = 0.02). This underscores engagement as a modifiable target to counteract aging-related synaptic loss, aligning with our 2-phase recovery framework: 8 weeks of BCI-FES jumpstarts neuroplasticity, while structured clinical rehabilitation (daily physical/cognitive therapy) preserves gains in the long term, a synergy supported by trials showing that adjunctive therapy is critical for sustained recovery [43]. Notably, our 8-week intervention duration balanced geriatric feasibility (compliance >90%) with neuroplastic timing: peak neural reorganization occurred within this window [44], matching meta-analyses confirming that intensive therapy (3 to 5 sessions/week) induces maximal cortical remodeling at 4 to 12 weeks, corresponding to our patients’ peak MoCA (17.0 at G4) and FMA-UE gains (19% improvement). Despite the potential benefits of extended protocols [45,46], fatigue tolerance in elderly cohorts limited session frequency, yet sustained motor recovery at G5 to G6 confirmed 8 weeks sufficed to initiate self-perpetuating plasticity when paired with ongoing rehabilitation.

Neurophysiologically, BCI-FES induced targeted neural remodeling in contralesional regions (C4, CP4, FC4, FT8, and T4), with marked reductions in DAR and DTABR accompanied by increased alpha/beta PSD and functional connectivity. These changes, absent in FES-only patients, reflect a precision mechanism: enhanced alpha/beta power fosters intra/interhemispheric communication, while delta suppression reduces pathological slow-wave activity, collectively optimizing small-world network efficiency (clustering coefficient +22%, local efficiency +18%, *P* < 0.05). PSD alterations strongly correlated with motor recovery (theta power at Cz predicted FMA-UE gains, *r* = 0.68), confirming that BCI-FES synchronizes cortical intent (via EEG) with peripheral activation (via FES) to reinforce Hebbian plasticity unlike open-loop FES, which elicits generalized, nonspecific responses. This closed-loop integration drove bilateral reorganization: ipsilesional alpha/beta synchronization (C3 and FC3) strengthened corticospinal pathways, while contralesional delta suppression (C4 and CP4) restored interhemispheric balance, mirroring functional MRI studies of compensatory recruitment in recovered movement.

Clinically, these neurophysiological shifts translated to superior outcomes: BCI-FES patients achieved 4.5 ± 1.2 points in FMA (Cohen’s *d* = 1.2, *P* < 0.01) versus 1.7 ± 0.8 in FES and 0.9 ± 0.6 in controls, with hemorrhagic stroke patients showing exceptional gains (median ΔFMA = 8, *P* < 0.01) and ischemic patients exhibiting higher variability (ΔFMA = 4.55 ± 2.1). Functional independence (MBI) improved by 5.4 ± 1.5 points (*d* = 1.1), with higher odds of reaching minimal clinically important differences (ΔMBI ≥ 12). Cognitive gains (MoCA Δ = 1.6 ± 0.5, *d* = 0.8 at G4) declined by G6, but engagement mitigated this: >65% MI accuracy correlated with 45.4% less cognitive decline (*P* = 0.02). Our predictive model identified key responders: age <70 years, stroke duration <23 months, and baseline MBI >40 predicted 76% of favorable outcomes, with an optimal treatment window of 3 to 12 months post-onset and 10 to 15 weeks of therapy (peak improvement index = 5.2).

We demonstrate that BCI-FES induces recovery in elderly stroke patients through 2 synergistic mechanisms: Hebbian plasticity-driven neural remodeling and corticospinal tract engagement, mediated by closed-loop sensorimotor integration. The intervention’s efficacy stems from its unique ability to synchronize cortical motor intent (detected via EEG) with peripheral neuromuscular activation (delivered via FES), creating a real-time feedback loop absent in conventional therapies. Unlike open-loop NMES/FES, which delivers passive stimulation without cortical engagement, BCI-FES reinforces temporally correlated activity between motor planning regions (ipsilesional sensorimotor cortex) and effector muscles, strengthening synaptic connections via Hebbian plasticity to drive bilateral reorganization. Crucially, despite substantial inter-individual variability in MI capacity, BCI-FES elicited comparable motor–cognitive gains across patients. This suggests that FES-enhanced proprioceptive feedback and not precise cortical decoding serves as the primary recovery driver. When combined with adaptive algorithms and paradigm switching, this mechanism ensures BCI-FES robustness against neurophysiological heterogeneity in elderly stroke populations.

Meta-analyses confirm that BCI interventions are effective across stages, with subacute application (<6 months) leveraging peak neuroplasticity during spontaneous recovery [47], showing 18% to 24% greater motor gains in non-elderly cohorts [48,49]. Mechanistically, early intervention could capitalize on heightened brain-derived neurotrophic factor expression [50,51] and prevent maladaptive plasticity consolidation [52]. For elderly patients specifically where our data prove retained chronic-phase plasticity (19% motor gain over 4.5 years), subacute BCI-FES might (a) amplify gains through earlier circuit remodeling, (b) mitigate accelerated aging-related decline via “neuroprotection by engagement”, (c) sculpt nascent neural circuits before synapse loss accelerates, and (d) reduce long-term disability by preventing maladaptive compensation. This represents a critical gap since no prior study has evaluated BCI-FES in both subacute/chronic elderly stroke with long-term follow-up.

In conclusion, BCI-FES drives sustained motor–cognitive recovery in elderly stroke patients via dual mechanisms: Hebbian plasticity from closed-loop sensorimotor integration and network optimization through targeted EEG biomarkers. Its efficacy, paired with predictive models of engagement and timing, provides a blueprint for personalized, aging-resilient rehabilitation, challenging the myth of irreparable aging brains and establishing BCI-FES as a transformative tool for neurorehabilitation.

## Materials and Methods

### Study design and populations

#### Subject recruitment and cohort characteristics

A total of 4,172 participants with stroke were initially screened from Huashan Hospital (Fudan University) and Shanghai Jinshan Zhongren Geriatric Nursing Hospital between January 2018 and December 2020, ensuring geographic and demographic diversity. Of these, 100 elderly stroke patients (60 to 90 years) were enrolled, with 24 chronic-stage subjects (>1 year poststroke onset) completing the full 4.5-year follow-up (BCI-FES: *n* = 12; FES-only: *n* = 12). The inclusion criteria were as follows: (a) diagnosis of ischemic/hemorrhagic stroke confirmed by computed tomography/MRI, (b) age 60 to 90 years, (c) stroke onset >1 month prior, (d) ability to sit independently for ≥1 h, and (e) with baseline FMA-UE scores ranging from 0 to 62. The exclusion criteria included severe aphasia, visual/hearing impairment, unilateral neglect, allergies to conductive paste, or comorbid neurological disorders (e.g., Parkinson’s disease). Baseline demographics (age, gender, stroke type, and lesion laterality) and clinical characteristics (pre-FMA, pre-MBI, and pre-MoCA) are summarized in Table S1, with balanced distribution across groups (χ^2^ test, *P* > 0.05 for all variables). The sample size calculations using GPower 3.1 based on FMA-UE effect sizes from prior BCI-FES trials [43,53] determined that 12 participants per group were required for longitudinal analysis (Cohen’s *d* = 0.75, *α* = 0.05, power = 0.8). The initial enrollment was 31 (BCI-FES), 35 (FES-only), and 34 (control) patients, with 24 chronic-stage subjects (12 per group) completing 4.5-year follow-up.

#### FES calibration

To optimize the BCI-FES strategy, the calibration method involves adjusting the signal generator to output a 200-μV, 10-Hz sine wave signal. The EEG acquisition system is then connected to the signal source in differential mode. The EEG software filtering parameters are configured as follows: the EEG low-pass filter is disabled (set to “None”), the 50-Hz notch filter is enabled, and the EEG sensitivity is set to 100 μV cm^−1^. A segment of data is recorded and then replayed to measure the sine wave amplitude. The per-channel gain values in the software’s “Calibration” module are adjusted until the measured amplitude reaches 200 μV, with an error tolerance of ≤5%. This calibration process ensures accurate signal responsiveness for effective integration of FES with BCI therapy. FES frequency parameters (20 to 50 Hz) were personalized for each patient through a sequential protocol to maximize efficacy and safety [54]. Real-time titration began at subthreshold intensities (5 to 10 mA), incrementally increasing by 2- to 5-mA steps until visible wrist dorsiflexion was achieved without discomfort (visual analog scale pain <3/10), with continuous electromyography monitoring to prevent pathological co-contraction. The session duration progressed from initial 5- to 10-min sessions to full 30-min treatments over 2 weeks, contingent on sustained torque output (>80% baseline) [43].

#### Intervention details

Both groups underwent routine rehabilitation therapy consisting of 1-h sessions of exercise and occupational therapy 5 times a week for a duration of 8 weeks. The BCI-FES group additionally participated in 40-min BCI-FES sessions 3 times weekly, while the FES group received 30-min FES sessions on the same schedule. FES was delivered using a commercial stimulator (Shanghai Nuo Cheng Electric Co., Ltd.) with biphasic pulses (frequency: 20 to 50 Hz; pulse width: 300 to 500 μs; amplitude: 10 to 30 mA). Electrodes were placed on the extensor carpi radialis and ulnaris muscles of the paretic limb. The stimulation intensity was adjusted to elicit visible wrist dorsiflexion without discomfort. Sessions included repetitive wrist extension/flexion tasks synchronized with therapist-guided movements to mimic functional tasks such as reaching and grasping. In the BCI-FES group, FES was activated through MI signals detected by the BCI system. Participants first completed familiarization sessions that incorporated real-time feedback and adaptive signal processing to ensure reliable engagement.

#### MI signal detection and calibration

The BCI-FES system (NCERP Series D EEG amplifier, Shanghai Nuo Cheng Electric Co., Ltd.) detected MI through C3/C4 EEG signals during visually cued hand movement trials. Signals were processed via common spatial patterns and linear discriminant analysis, with sensorimotor rhythm bands personalized per patient (alpha: 8 to 12 Hz; beta: 18 to 24 Hz) to maximize discriminability [55]. A dynamically adjusted threshold initially 70% of the maximum classifier output (dynamic range: 55% to 85%) triggered FES upon MI detection, modified weekly: increased by 5% if the accuracy exceeded 75% for 2 sessions, decreased by 5% if the accuracy fell below 60%, or maintained for 60% to 75% accuracy. Real-time error correction increased independent-component-analysis-based artifact rejection sensitivity for false positives and expanded frequency bands (e.g., alpha: 7 to 14 Hz) for false negatives. Throughout 50-trial sessions (including 10 calibration trials), visual feedback (virtual hand proportional to MI strength) and adaptive neurofeedback reinforced engagement, with classification stability confirmed by high test–retest reliability.

#### Rehabilitation assessment

Patients were evaluated at 6 distinct time points: pre-intervention (G1), 1 month post-intervention (G2), 2 months post-intervention (G3), 4 months post-intervention (G4), 2 years post-intervention (G5), and 4.5 years post-intervention (G6). Motor recovery was assessed through FMA-UE (arm/hand mobility), cognitive recovery was assessed via MoCA (memory, attention, and language), and activities of daily living were assessed by MBI (self-care and mobility). These evaluation tools were selected for their established reliability and sensitivity to changes in motor, cognitive, and functional recovery post-rehabilitation.

### EEG processing

#### EEG recording and preprocessing

Resting-state brain activity was recorded using a 24-electrode cap positioned according to the international 10–20 system, covering frontal (F3, F4, Fz, FC3, FC4, FCz, FT7, and FT8), central (C3, C4, and Cz), parietal (CP3, CP4, P3, P4, CPz, and Pz), temporal (T3, T4, TP7, and TP8), and reference regions (A1, A2, and Oz). Patients underwent two 5-min eyes-closed recordings pre- and post-intervention. To minimize fatigue effects validated by established EEG protocols showing terminal-phase attentional drift with increased theta/delta power and decreased alpha coherence [56], the final minute of each recording was excluded. This approach was empirically confirmed in our cohort, where the terminal minute exhibited significant delta power increase (+18.7%, *P* = 0.01) and alpha decrease (−12.3%, *P* = 0.03) versus initial segments, and clinically justified by accelerated fatigue in elderly stroke patients during static tasks [57]. The remaining 8 min of data per session were divided into four 2-min epochs, then filtered (0.5- to 60-Hz bandpass), re-referenced to a common average, and cleaned via automated artifact removal (ocular/muscular artifacts) and 50-Hz notch filtering.

#### EEG biomarkers

To evaluate neurophysiological changes, biomarkers such as PSD, relative power spectral density (rPSD), DAR, DTABR, and pairwise-derived brain symmetry index (pdBSI) were analyzed and detailed in the Supplementary Methods. These metrics provided insight into frequency-specific changes, interhemispheric symmetry, and oscillatory balance linked to motor and cognitive recovery.

#### Power spectral density

As a quantitative EEG measure, PSD provides insight into the distribution of neuronal oscillation power across frequency bands, which has been shown to correlate with poststroke recovery outcomes [58]. The total power in delta (0.5 to 4 Hz), theta (4 to 8 Hz), alpha (8 to 13 Hz), and beta (13 to 30 Hz) bands reflects regional brain activation. PSD was calculated for 10-min clean EEG recordings using the Welch method. A rectangular window with a length of 20 s and a 5-s overlap was applied to improve spectral estimation stability. The PSD values for each channel were averaged across all rectangular windows to compute a robust estimate of power. The global PSD was then obtained for each frequency bin (with a resolution of 0.125 Hz) by averaging across all 22 channels.

#### Relative PSD

rPSD was calculated by normalizing the PSD within each frequency band relative to the total power in the 1- to 60-Hz range. This normalization provides a clearer understanding of the relative contribution of each frequency band to brain activity, eliminating potential biases from intersubject variability in absolute power. Global rPSD values were derived by averaging across all 22 channels.

#### Delta/alpha power ratio

The ratio of delta (0.5 to 4 Hz) to alpha (8 to 13 Hz) power is used to evaluate brain dysfunction, with a lower DAR indicating better brain function.

#### (Delta + theta)/(alpha + beta) power ratio

The (delta + theta)/(alpha + beta) power ratio is a neurophysiological biomarker that quantifies the balance between low-frequency (delta: 0.5 to 4 Hz; theta: 4 to 8 Hz) and high-frequency (alpha: 8 to 13 Hz; beta: 13 to 30 Hz) brain oscillations. A lower DTABR value indicates a shift toward higher-frequency activity, reflecting improved cortical excitability, reduced pathological slow-wave activity, and enhanced neuroplasticity.

#### Pairwise brain symmetry index

The hemispheric symmetry index is calculated as a measure of interhemispheric balance, derived from the absolute difference in PSD between homologous electrode channels in the left and right cerebral hemispheres. Lower pdBSI values indicate greater symmetry, associated with recovery (lower pdBSI = greater symmetry = better recovery). Detailed calculations of DTABR and pdBSI can be found in the Supplementary Methods.

#### Brain network analysis

Functional connectivity was mapped by measuring synchronization between brain regions (pairwise phase consistency). Networks were modeled as graphs: nodes = EEG channels and edges = synchronized connections. Networks were thresholded to remove spurious connections, and graph theory metrics including node strength, clustering coefficient, local efficiency, global efficiency, characteristic path length, and small-world index were computed to assess network topology. Node strength refers to the importance of specific hubs that are highly connected and play a critical role in facilitating communication between other nodes. Strong node strength indicates that a hub can influence and manage a large amount of information flow, thus supporting efficient network functioning and integration across different brain regions. Clustering coefficient/local efficiency reflects the efficiency of local information processing within a network. A high clustering coefficient indicates that nodes tend to cluster together, facilitating rapid exchanges of information among closely connected nodes. Local efficiency measures how well information is transmitted within a specific area of the network, highlighting the brain’s ability to process information quickly and effectively within localized regions. Global efficiency assesses the overall efficiency of long-range communication within a network, indicating how quickly information can be transmitted across the entire network. Path length measures the average number of steps required to connect 2 nodes, with shorter paths allowing for faster communication. High global efficiency suggests that the network is well organized for swift information transfer, crucial for coordinated brain function. Small-world index represents the balance between local and global efficiency within a network. A high small-world index indicates an optimal network topology where there are many local connections (facilitating effective localized processing) and a few long-range connections (ensuring rapid communication across the network). This balance is essential for maintaining flexibility and robustness in neural communication, enhancing both functional recovery and adaptability in cognitive processes.

### Predictive modeling of treatment response

#### Data preprocessing

Clinical and demographic data were preprocessed using Python 3.8 (pandas 1.4.2) to handle missing values (multiple imputation via MICE, 5 iterations) and normalize features (z-score transformation for age and stroke onset months). Key predictors included age, stroke duration (months), pre-FMA, pre-MBI, and stroke type (ischemic/hemorrhagic). The primary outcome was FMA improvement (ΔFMA = post-FMA − pre-FMA).

#### Model development

A random forest regression model was constructed to predict ΔFMA using scikit-learn 1.0.2. Hyperparameters were optimized via 5-fold cross-validation (GridSearchCV) with the following search space: *n_estimators* (50 to 200), *max_depth* (3 to 10), and *min_samples_split* (2 to 10). The model was trained on 80% of the data (stratified by stroke type) and validated on the remaining 20%, with performance metrics including *R*^2^, mean absolute error, and root mean squared error.

#### Code availability

The complete Python code for data preprocessing, model training, and visualization is provided in the Supplementary Code.

#### Statistical analysis

Continuous variables are presented as mean ± SD or median (IQR) based on normality (Shapiro–Wilk test). Group comparisons were performed using one-way analysis of variance or Kruskal–Wallis tests, with post hoc Bonferroni correction. Correlations between EEG biomarkers and clinical outcomes were assessed via Pearson’s or Spearman’s rank coefficients. All statistical analyses were conducted in R 4.1.2 (packages: dplyr, ggplot2, and lme4) and Python (scipy 1.8.0), with significance set at *P* < 0.05.

## Ethical Approval

This study was approved by the Ethics Committee of Huashan Hospital (KY2014-266) and conducted by the Declaration of Helsinki (https://www.wma.net/policies-post/wma-declaration-of-helsinki/), with informed consent obtained from all participants.

## Data Availability

The datasets generated and analyzed during the current study, including neuropsychological score maps, EEG biomarkers, and brain network data, are available in the supplementary files. Processed data and specific analysis scripts used for result generation can be requested from the corresponding authors upon reasonable request. All neuropsychological data from the included elderly stroke patients can be accessed through the Huashan Hospital’s institutional repository (PId, 893; https://himedc.huashan.org.cn:5288/redcap_v999.0.0/).

## Supplementary Materials

Supplementary 1 Supplementary Methods Tables S1 to S3 Figs. S1 to S13 Video S1 Supplementary Data of Cohort Supplementary Code
