# Supplementary material for: Long-Term Brain–Computer Interface Functional Electrical Stimulation Enhances Neuroplasticity and Functional Recovery in Elderly Stroke: A 4.5-Year Longitudinal Study Integrating Electroencephalography Biomarkers and Clinical Assessments
Source: Research (Wash D C). 2025 Dec 4;8:0984. doi: 10.34133/research.0984 (PMC12677916; doi:10.34133/research.0984)
Supplement: Supplementary 1 — Supplementary Methods Tables S1 to S3 Figs. S1 to S13 Video S1 Supplementary Data of Cohort Supplementary Code [file research.0984.f1.zip › Supplementary materials.pdf]

Supplementary Information

Long-Term BCI Functional Electrical Stimulation Enhances Neuroplasticity and Functional Recovery in Elderly Stroke: A 4.5-Year Longitudinal Study Integrating EEG Biomarkers and Clinical Assessments

Shugeng Chen[1,†], Na Xie[2,†], Yurui Tang[1,†], Yanyun Ji[3], Zhijie He[1], Yuchun Wang[1], Xude Huang[1], Jianghong Fu[1], Minyan Ge[1], Qiang Liu[1], Mingfen Li[4], Qinqin Xiao[3], Ying Xu[3], Jing Wang[1,*], Jie Jia[1,5,6*], Shumao Xu[1,*]*

[1]Department of Rehabilitation Medicine, Huashan Hospital, Institute of Science and Technology for Brain-inspired Intelligence (ISTBI), Fudan University, Shanghai, 201203, China

[2]Peking University International Hospital, Peking University Eighth School of Clinical Medicine, Beijing, 102206, China

[3]Shanghai Jinshan Zhongren Geriatric Nursing Hospital, Shanghai, 201501, China

[4]Department of Neurorehabilitation, Hubei Provincial Clinical Research Center for central nervous system repair and functional reconstruction, Taihe Hospital, Hubei University of Medicine, Hubei, 422000, China

[5]National Center for Neurological Disorders, Shanghai, 200040, China

[6]National Clinical Research Center for Aging and Medicine, Huashan Hospital, Fudan University, Shanghai, 200040, China

[†]These authors have contributed equally to this work.

[*]Correspondence: tonychshug@126.com (S.C.); wangjing_@fudan.edu.cn (J.W.); shannonjj@126.com (J.J.); shumaoxu@fudan.edu.cn (S.X.).

Supplementary Methods

Functional electrical stimulation (FES) for rehabilitation training

FES is employed as pulse current therapy aimed at upper extremity rehabilitation. Each session lasts for 30 minutes and is conducted three times per week. The stimulation intensity is customized for each patient, determined by their hand responses to ensure both comfort and effectiveness. The parameter for FES was set to produce observable muscle contractions conducive to rehabilitation while avoiding discomfort for the user. In conjunction with BCI therapy, the total duration of treatment extends to 40 minutes. For BCI-FES, 10 min were allocated to EEG setup/calibration. The remaining 30 min involved identical FES-assisted motor training as the FES group. This integrated approach utilizes FES to facilitate motor activities in response to cortical signals associated with movement intent. Electrodes are strategically placed on the forearm and secured using a highly conductive electrolyte spray to enhance stimulation effectiveness. A pulse rate of 20-50 Hz is used to generate tetanic contractions. Stimulation intensity begins at zero and can be adjusted in increments of 0.5 mA, prioritizing participant comfort throughout the process. Should any discomfort arise, the intensity is promptly reduced to the nearest tolerated level. The design is to achieve notable muscle contractions, either flexion or extension of the fingers, ensuring that the output does not exceed 50 mA.

Rehabilitation therapy in the control group

Patients in the control group received conventional rehabilitation therapy. This standard clinical intervention included: 1) physical therapy (incorporating techniques such as stretching and task-oriented limb training etc., 20 minutes per session), 2) occupational therapy (20 minutes per session), and 3) functional hand training (20 minutes per session). Treatments were administered five times per week, with a total duration of 60 minutes per session. Consistency in rehabilitation therapists was maintained wherever feasible throughout the study period.

EEG recording and preprocessing

The EEG cap was utilized for capturing brain activity associated with movement intent in participants undergoing the BCI-FES intervention. Each participant is equipped with an EEG cap that adheres to the standard 10-20 international electrode placement system, ensuring consistent and optimal positioning of 24 electrodes across the scalp. The electrodes are strategically located over the sensorimotor cortices, specifically targeting the C3, C4, and Cz regions, to record relevant electrical activity. To facilitate signal acquisition, the EEG cap is grounded to the Fpz location and referenced to an electrode positioned at the back of the participant's right ear. This configuration enhances the signal quality by properly referencing the acquired data. The electrodes utilize sintered Ag/AgCl to achieve high fidelity in recording, capturing subtle changes in electrical activity during motor tasks.

During the recording sessions, EEG signals are digitally captured through a 24-channel bipolar recording system, which is designed to maintain a high signal-to-noise ratio. The EEG signals undergo amplification via specialized equipment to ensure that even the smallest voltage changes are detected clearly. Data acquisition occurs at a sampling rate of 512 Hz with data segmented into four epochs (epochs 1-4) for detailed analysis, enabling detailed analysis of brain activity, with a band-pass filter set between 0.1 to 100 Hz to eliminate noise from irrelevant frequency ranges. Reference signals from A1 and A2 were excluded prior to preprocessing. Band-pass filtering was applied to extract signals in the delta (0.5-4 Hz), theta (4-8 Hz), alpha (8-13 Hz), beta (13-30 Hz), and gamma (30-60 Hz) bands. The BCI2000 platform is utilized to process these signals. This involves real-time spectral analysis, where the system calculates the power spectrum of the signals. The processing includes an autoregressive modeling approach which captures the spectral amplitude and translates it into control features for cursor movement. Throughout the BCI-FES tasks, the EEG signals serve as input for translating user intent into

actionable movements within the virtual environment. The neural data captured from the specified channels and frequency bands provides objective feedback, directly influencing the efficacy of the intervention and supporting the reinforcement of motor learning through multimodal feedback mechanisms.

EEG features

In this study, the calculation of EEG biomarkers was designed to capture variations across different frequency bands and temporal segments to explore neurophysiological changes induced by the interventions. EEG data were segmented into five frequency bands (delta, theta, alpha, beta, gamma) and four temporal epochs (epochs 1-4), allowing for a detailed temporal and spectral analysis. This stratification aimed to provide a high-resolution understanding of potential changes in brain networks and activity patterns associated with functional outcomes.

Specifically, power spectral density (PSD) and relative power spectral density (rPSD) were calculated to measure the magnitude of neuronal oscillations within these frequency bands. The delta/alpha power ratio (DAR) and (theta + delta)/(alpha + beta) power ratio (DTABR) was computed to examine broader patterns of low-to-high frequency oscillatory balance, which are known to reflect motor and cognitive recovery mechanisms. Additionally, the brain symmetry index (BSI) was used to assess the hemispheric symmetry of neural activity across all 22 electrodes, providing insights into the spatial distribution of recovery-related activity. To establish potential links between these changes and functional connectivity, we complemented the PSD and rPSD analysis with brain network measures derived from graph theory. The integration of these analyses aimed to correlate spectral changes with specific connectivity patterns and neuroplasticity mechanisms.

DAR calculation:

$$\mathrm{DAR}_c = \frac{\langle P_c(f) \rangle_{f=0.5}^{4}}{\langle P_c(f) \rangle_{f=8}^{13}} \qquad (1)$$

where $P_c(f)$ is the absolute power at frequency *f* for channel *c*. Global DAR is averaged across all channels, *N* (*N* is equal to 22 in this work):

$$\mathrm{DAR} = \frac{1}{N} \sum_{c=1}^{N} \mathrm{DAR}_c \qquad (2)$$

DAR indicates the balance between slow (delta) and fast (alpha) oscillations, reflecting cortical activity.

DTABR calculation:

$$DTABR_c = \frac{P_\delta + P_\theta}{P_a + P_\beta} \qquad (3)$$

where P_δ, P_θ, P_a, and P_β are the powers in respective bands for channel *c*. Global DTABR is computed as:

$$DTABR = \frac{1}{N} \sum_{c=1}^{N} DTABR_c \qquad (4)$$

DTABR indicates that low-frequency oscillations are more prominent than higher-frequency activity in the brain. During specific mental states or tasks related to EEG-FES stroke recovery, the amplitude of low-frequency waves, which are associated with deep relaxation and memory processes, surpasses that of higher-frequency waves, which are typically linked to alertness and cognitive engagement.

pdBSI calculation:

$$pdBSI = \frac{1}{NM} \sum_{j=1}^{M} \sum_{i=1}^{N} \left| \frac{R_{ij} - L_{ij}}{R_{ij} + L_{ij}} \right| \qquad (5)$$

where R_{ij} and L_{ij} respectively were the power spectral density using Welch's method of the EEG signal obtained from the right and left channels of a homologous channel pair (i=1,2, … , M) at frequency j (j=1,2, … , N). M, the number of homologous channel pairs, was set to 8 in this study (F3-F4, FC3-FC4, C3-C4, CP3-CP4, P3-P4, FT7-FT8, T3-T4, TP7-TP8), and N represents the frequency range analyzed across five bands: delta (0.5-4 Hz), theta (4-8 Hz), alpha (8-13 Hz), beta (13-30 Hz), and gamma (30-60 Hz). This pdBSI calculation quantified symmetry across frequency bands and homologous channel pairs, providing a detailed view of interhemispheric balance.

Potential differences in baseline EEG asymmetry, which could arise from lesion location, size, or type, were addressed to ensure the validity of the results. Baseline pdBSI values were calculated for all participants before the intervention, and these values were stratified based on lesion laterality (left vs. right hemisphere). Homologous channel pairs were analyzed individually to account for any pre-existing asymmetries due to stroke location. To further minimize the influence of baseline inter-group asymmetry, pdBSI changes were analyzed as relative differences (ΔpdBSI) by subtracting pre-intervention values from post-intervention values. This normalization isolated the effects of the intervention from pre-existing asymmetry.

Within each group, the potential impact of baseline asymmetry on pdBSI trends was assessed by performing subgroup analyses based on lesion type (cortical vs. subcortical stroke) and severity (as measured by initial motor scores). Statistical testing confirmed that baseline asymmetries did not significantly differ between groups, reducing the likelihood of confounding effects. Observed pdBSI changes were related to neuroplasticity. Reductions in asymmetry in the alpha and beta bands across motor-related homologous pairs (C3-C4, CP3-CP4) were consistent with functional recovery and interhemispheric coordination post-intervention. Similarly, significant reductions in asymmetry in the delta band, primarily observed in the BCI-FES group, aligned with the suppression of pathological slow-wave activity, suggesting that these changes reflected therapeutic effects rather than pre-existing disparities. By controlling baseline asymmetry and focusing on relative pdBSI changes, the study minimized the influence of lesion-specific factors on the observed inter-group and intra-group EEG results, which ensured that the reported findings accurately reflected the effects of BCI-based interventions and provided robust insights into the dynamic interplay between hemispheric reorganization and functional recovery.

Brain network analysis

Based on the preprocessed EEG data, functional connectivity brain networks were constructed for both the BCI-FES and FES groups before and after the intervention by calculating PPC. Individual EEG channels were defined as nodes, resulting in brain networks with 22 nodes corresponding to the 22 EEG channels. Following the PPC calculations, two 22 × 22 weighed connection matrices were generated for each subject, representing connectivity before and after the intervention. To eliminate potential spurious connections, proportional thresholding was applied to the weighted connection matrices, with the threshold set to 0.3 (determined through density-based optimization to preserve network sparsity). Graph theory metrics, including node strength, clustering coefficient, local efficiency, global efficiency, characteristic path length, and small-world index, were calculated to comprehensively evaluate the topological differences in brain networks within and between groups. To provide a clear visualization of brain network connections, weighted connection matrices were averaged across all subjects within each group, yielding average weighted connection matrices. In these functional connectivity brain networks, the size and color of the nodes represent normalized node strength, while the connections between node pairs indicate the presence of a relationship rather than the weight of the connection.

In an undirected weighted network, the node strength of node i is defined as:

$$NS_i = \sum_j w_{ij} \qquad (6)$$

where w_{ij} is the connection weight between node i and node j. This equation indicates node importance in network communication.

Statistical analysis of EEG features

To assess the differences between the BCI-FES group and the FES group before and after the intervention, we performed statistical analyses on five biomarkers. We evaluated the normality of the variables using Shapiro-Wilk tests, which revealed that they did not follow a normal distribution. Consequently, non-parametric tests were implemented. For intra-group comparisons before and after the intervention, paired sample Wilcoxon signed-rank tests were applied. To determine if there was a statistically significant difference between the two groups, Mann-Whitney U tests were conducted. All statistical tests were based on a significance level of $p < 0.05$.

Considering the multidimensional nature of the data, we examined the significant changes before and after the intervention for the same treatment approach. We focused on the motor-related C3/C4 channels and their surrounding eight channels (FC3, FT7, TP7, CP3, FC4, FT8, TP8, CP4). These areas were considered due to their relevance to motor function and potential implications in stroke recovery. To eliminate the interference of baseline inequality, we calculated the difference between pre- and post-intervention values within groups and statistically analyzed the differences. The Shapiro-Wilk parametric hypothesis test was first used to test whether the samples satisfy normal distribution. For samples satisfying normal distribution, differences between groups were assessed using an independent two-sample *t*-test. The nonparametric test we used to test for between-group differences was the Wilcoxon rank sum test. $p < 0.05$ was used as the criterion for a statistically significant difference in the test.

Sobel test

Sobel test is a statistical method used to assess whether a mediator variable significantly explains the relationship between an independent variable (X) and a dependent variable (Y). It evaluates whether the indirect effect (the pathway through the mediator) is statistically significant.
Mediation analysis: Tests if the effect of X on Y operates through a third variable (M).
Direct effect: X → Y
Indirect effect: X → M → Y
Sobel test formula:

$$z = \frac{a \times b}{\sqrt{b^2 \times SE_a^2 + a^2 \times SE_b^2}}$$

where a = coefficient of X → M; b = coefficient of M → Y (controlling for X); SE_a, SE_b = standard errors of a and b. A significant result implies M meaningfully transmits X's effect on Y.

In this work, independent variable (X): BCI-FES intervention;
Mediator (M): Patient engagement (composite score: task adherence + EEG theta power + VAS fatigue);
Dependent Variable (Y): Long-term motor recovery (ΔFMA-UE at 4.5 years);
Indirect effect (a×b): 0.47 (47% mediation); p-value: 0.01;
Standard errors: SE_a (X→M path): 0.18; SE_b (M→Y path): 0.12
Path coefficients: a (X→M): 0.63 ($p < 0.001$); b (M→Y): 0.41 ($p = 0.008$)

Calculation: $z = \frac{a \times b}{\sqrt{b^2 \times SE_a^2 + a^2 \times SE_b^2}} = \frac{0.63 \times 0.41}{\sqrt{(0.41)^2 \times (0.18)^2 + (0.63)^2 \times (0.12)^2}} = \frac{0.2583}{\sqrt{0.005476 + 0.005734}} = \frac{0.2583}{\sqrt{0.01121}} = \frac{0.2583}{0.106} = 2.44$. The mediation is highly significant (|z|=2.44 > 1.96; *p*=0.01), confirming engagement is a non-random mechanistic driver.

Supplementary Tables

Supplementary Table 1. Characteristics of demographics of 24 patients during the follow up

Patient	Gender	Age (years)	Diagnosis	Lesion site	Lesion side	Time since stroke (month)	Pre FMA-UE (G1)	Post FMA-UE (G3)
**BCI-FES group**								
BCI01	man	77	Ischemic	Cortical & subcortical	right	34	3	5
BCI02	man	75	Ischemic	Cortical	right	19	1	5
BCI03	man	82	Ischemic	Subcortical	left	74	25	28
BCI04	man	68	Ischemic	Cortical	right	12	12	12
BCI05	man	63	Ischemic	Subcortical	right	39	5	7
BCI06	man	67	Hemorrhagic	Cortical	right	12	4	6
BCI07	man	84	Ischemic	Cortical	right	145	32	35
BCI08	female	81	Ischemic	Cortical & subcortical	right	26	33	33
BCI09	man	83	Ischemic	Cortical & subcortical	left	14	52	53
BCI10	man	75	Hemorrhagic	Cortical & subcortical	right	16	30	30
BCI11	man	68	Ischemic	Cortical & subcortical	right	29	30	30
BCI12	man	72	Ischemic	Cortical & subcortical	right	40	3	5
**FES group**								
FES01	man	82	Ischemic	Cortical	left	27	0	0
FES02	man	86	Ischemic	Subcortical	right	12	58	58
FES03	man	84	Ischemic	Subcortical	right	28	45	45
FES04	man	77	Ischemic	Subcortical	right	22	27	25
FES05	man	68	Ischemic	Cortical & subcortical	right	41	20	20
FES06	man	86	Ischemic	Subcortical	left	36	44	43
FES07	man	80	Ischemic	Cortical	left	44	2	2
FES08	man	76	Hemorrhagic	Subcortical	left	110	6	6
FES09	man	78	Ischemic	Cortical & subcortical	right	35	56	59
FES10	man	76	Ischemic	Cortical	left	180	31	30
FES11	man	83	Ischemic	Cortical & subcortical	left	30	24	24
FES12	female	73	Ischemic	Cortical & subcortical	left	32	30	30

FES, functional electrical stimulation; FMA-UE, Fugl-Meyer assessment scale of the upper extremity.

Supplementary Table 2. **Baseline demographics, intervention parameters, and longitudinal effects of BCI-FES in elderly stroke patients**

**Category**	**Details**				
**Baseline demographics**					
Sample size	24 chronic stroke patients				
Age range (mean)	60–90 years (mean 77 ± 5.2)				
Gender	91.7% male, 8.3% female				
Time since stroke	>1 year (chronic phase)				
Affected side	37.5% left hemisphere, 62.5% right hemisphere				
**Intervention parameters**					
Therapy duration	8 weeks (3 sessions/week)				
Session length	40 minutes/session				
BCI protocol	EEG-based motor imagery detection (24 channels: C3, C4, FC3, FC4, etc.)				
FES activation	Synchronized with motor intent (wrist dorsiflexors; 20–40 mA, 300 μs pulses)				
Follow-up period	4.5 years (assessments at 1 month, 2 months, 4 months, 2 years, 4.5 years post-intervention)				
**Longitudinal effect sizes**					
Clinical metrics	1 month	2 months	4 months	2 years	4.5 years
FMA-UE (motor)	Δ +1.8*	Δ +3.1**	Δ +3.5**	Δ +3.7** (19%)	Δ +3.2*
MBI (daily living)	Δ +4.2*	Δ +6.5**	Δ +7.1**	Δ +8.0 (22%)	Δ +6.8**

Category	Details				
MoCA (cognition)	Cohen's *d* = 0.41	Cohen's *d* = 0.76**	Cohen's *d* = 0.68*	Cohen's *d* = 0.53	Cohen's *d* = 0.31
**Neurophysiological metrics**	1 month				
Alpha power ($\mu V^2/Hz$)	+12%*				
Delta power ($\mu V^2/Hz$)	-9%*				
pdBSI (symmetry index)	-0.06*				
Small-world index	+0.07*				

Statistical significance: $p < 0.05$; *$p < 0.01$; **$p < 0.001$ (paired t-test vs. baseline).

FMA-UE: Fugl-Meyer assessment (upper extremity); MBI: Modified Barthel index; MoCA: Montreal cognitive assessment; pdBSI: Pairwise-derived brain symmetry index.

Supplementary Table 3. Improvements in scales

		Before VS after（BCI-FES group）					BCI-FES group vs FES group					
		G1&G2	G1&G3	G1&G4	G1&G5	G1&G6	G1	G2	G3	G4	G5	G6
FMA	Cohen's *d*	-0.097	-0.097	-0.170	-0.231	-0.235	-0.517	-0.428	-0.428	-0.324	-0.061	-0.020
	effect size	-0.048	-0.048	-0.085	-0.115	-0.116	-0.250	-0.209	-0.209	-0.160	-0.030	-0.010
MoCA	Cohen's *d*	-0.338	-0.338	-0.472	-0.198	-0.099	-0.105	0.287	0.247	0.756	0.680	0.902
	effect size	-0.167	-0.167	-0.223	-0.099	-0.049	-0.052	0.142	0.122	0.353	0.322	0.411
MBI	Cohen's *d*	-0.299	-0.371	-0.344	0.144	-0.158	0.164	0.339	0.372	0.513	0.189	0.699
	effect size	-0.148	-0.183	-0.169	0.072	-0.079	0.082	0.167	0.183	0.248	0.094	0.330

Differences in data were analyzed between post-intervention stages (G2, G3, G4, G5, G6) and the pre-intervention baseline (G1) within the BCI-FES group, as well as across all six stages (G1, G2, G3, G4, G5, G6) between the BCI-FES and FES groups. Cohen's d and effect sizes for the three scales (FMA, MoCA, MBI) are detailed in Table 2. Stages are defined as follows: G1, before intervention; G2, 1 month after intervention; G3, 2 months after intervention; G4, 4 months after intervention; G5, 2 years after intervention; G6, 4.5 years after intervention.

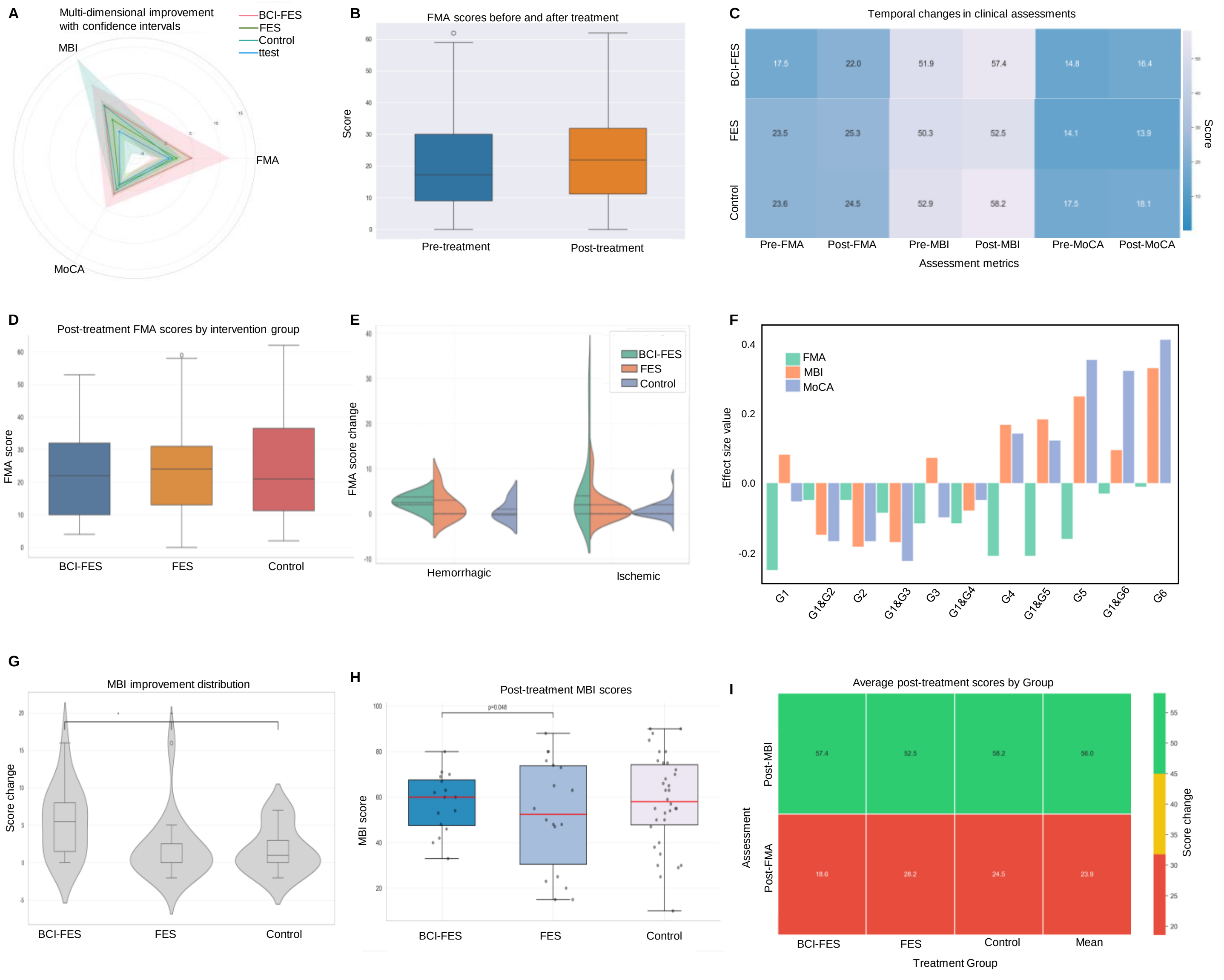

Supplementary Figure 1. Multidimensional clinical outcomes of BCI-FES, FES, and control interventions in neurorehabilitation. (A) Radar chart showing multidimensional improvements with 95% confidence intervals, demonstrating broader gains in BCI-FES and FES groups versus control. **(B)** Box plots of FMA scores pre- and post-treatment, highlighting increased motor function across all groups post-intervention. **(C)** Heatmap of temporal changes in clinical metrics, with BCI-FES showing the most consistent pre-to-post improvement. **(D)** Post-treatment FMA scores by intervention group, with BCI-FES and FES groups outperforming control. **(E)** Density plots of FMA score distribution stratified by stroke type (hemorrhagic/ischemic), revealing larger post-treatment score shifts in BCI-FES for both subtypes. **(F)** Effect sizes for FMA, MBI, and MoCA, indicating positive treatment effects for BCI-FES and FES across outcomes. **(G)** Violin plots of MBI improvement distribution, showing greater variability and higher median gains in BCI-FES compared to FES and Control. **(H)** Scatter plots of post-treatment MBI scores with group means, demonstrating clustering of higher scores in BCI-FES. **(I)** Bar chart of average post-treatment scores, confirming BCI-FES as the most effective intervention for improving MBI, FMA, and MoCA, followed by FES and control.

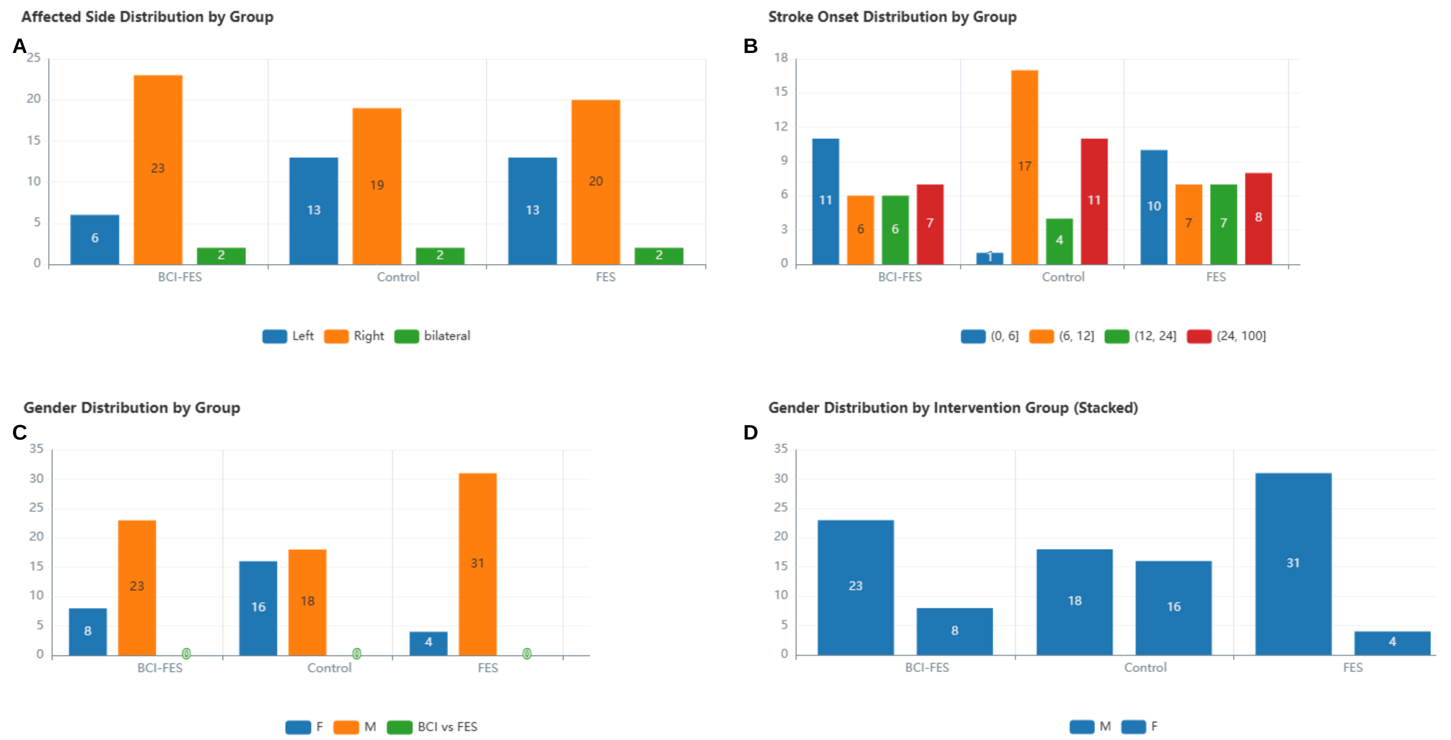

Supplementary Figure 2. Baseline demographic and clinical characteristics across study groups. (**A**) Affected side distribution by group. All three identical charts show the distribution of left-, right-, and bilaterally affected sides. (**B**) Stroke onset distribution by group. Stroke onset time is categorized into <6, 6–12, 12–24, 24–72, and >72 months. (**C**) Gender distribution by group. Gender varies by group: BCI+FES has more females than males. (**D**) Gender distribution by intervention group.

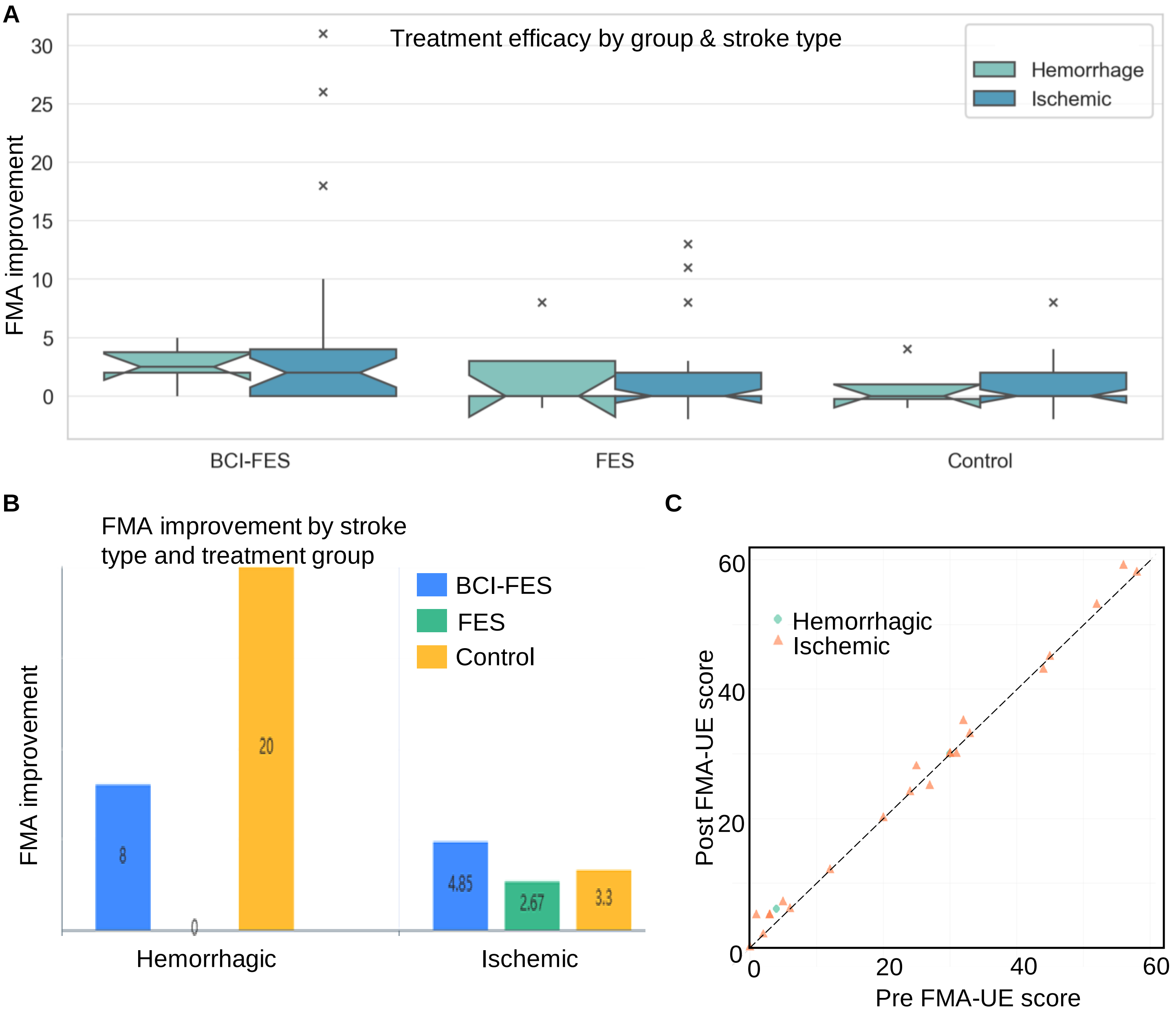

Supplementary Figure 3. Comparison of functional motor recovery (FMA improvement) across stroke types and treatment groups. (A) Box plots showing Fugl-Meyer assessment (FMA) improvement scores stratified by treatment group (BCI-FES, FES, Control) and stroke type. Data points (×) represent individual patient values. BCI-FES and FES groups demonstrate higher median FMA improvement compared to the Control group, with ischemic stroke patients generally showing greater variability in response. **(B)** Bar graph summarizing mean FMA improvement by stroke type and treatment. **(C)** Scatter plot with trendlines illustrating the relationship between pre-treatment and post-treatment FMA-UE scores for hemorrhagic and ischemic stroke patients. A strong positive correlation is observed for both stroke types, with ischemic patients generally achieving higher post-treatment scores across baseline values.

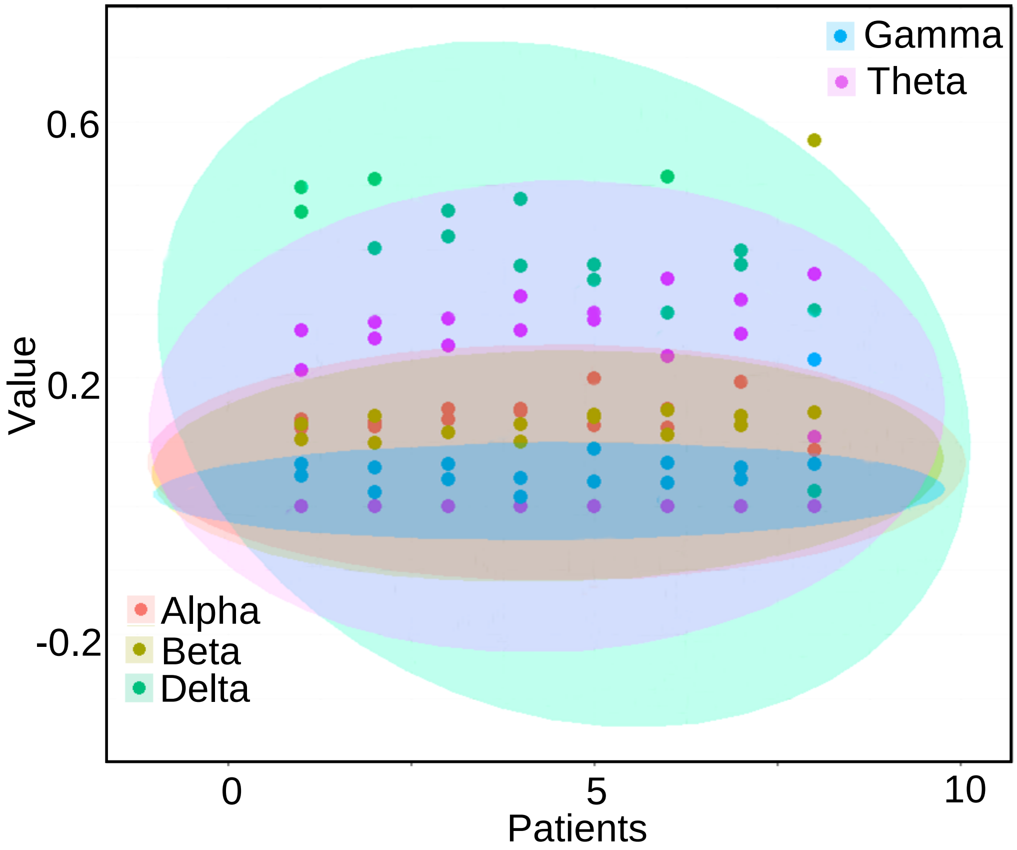

Supplementary Figure 4. Multidimensional plot demonstrating the association of EEG frequency bands (gamma, theta, alpha, beta, delta) with post-intervention recovery across patients.

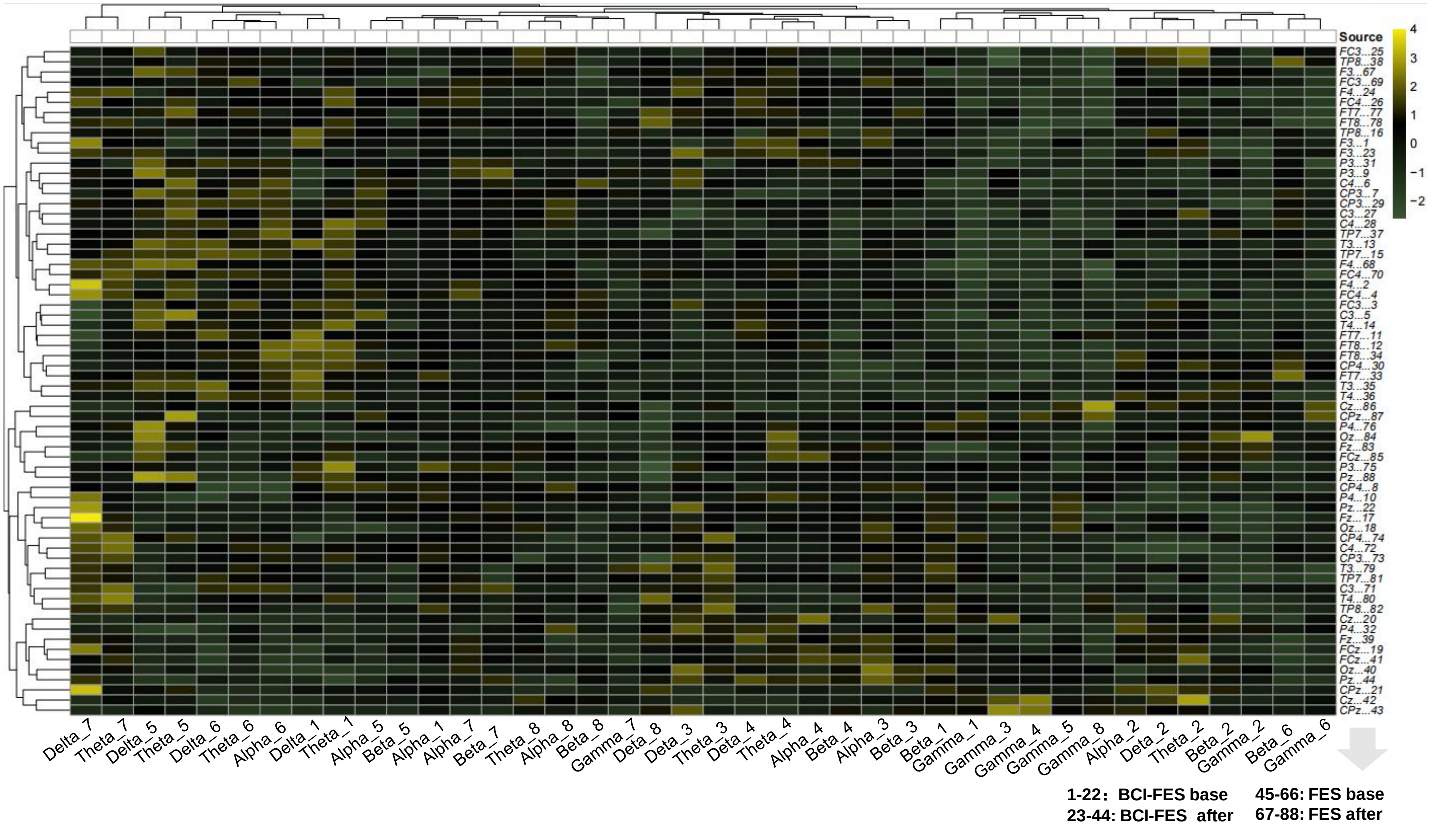

Supplementary Figure 5. Heatmap analysis of EEG channel activity across BCI-FES and FES interventions. Hierarchical clustering represented by a heatmap displaying the relative activity of different EEG channels (FC3, TP8, *etc*.) across patient samples (*x*-axis), categorized by intervention type without and with functional electrical stimulation after 4.5-year follow-up. The *y*-axis lists EEG channel locations (1-22 for BCI-FES baseline, 23-44 for BCI-FES after 4.5 years, 45-66 for FES baseline, and 67-88 for FES after 4.5 years). The color gradient indicates normalized activity levels, with yellow representing higher activity and black denoting lower activity. This heatmap highlights alterations in channel activity associated with the BCI-FES and FES therapeutic interventions over the treatment duration.

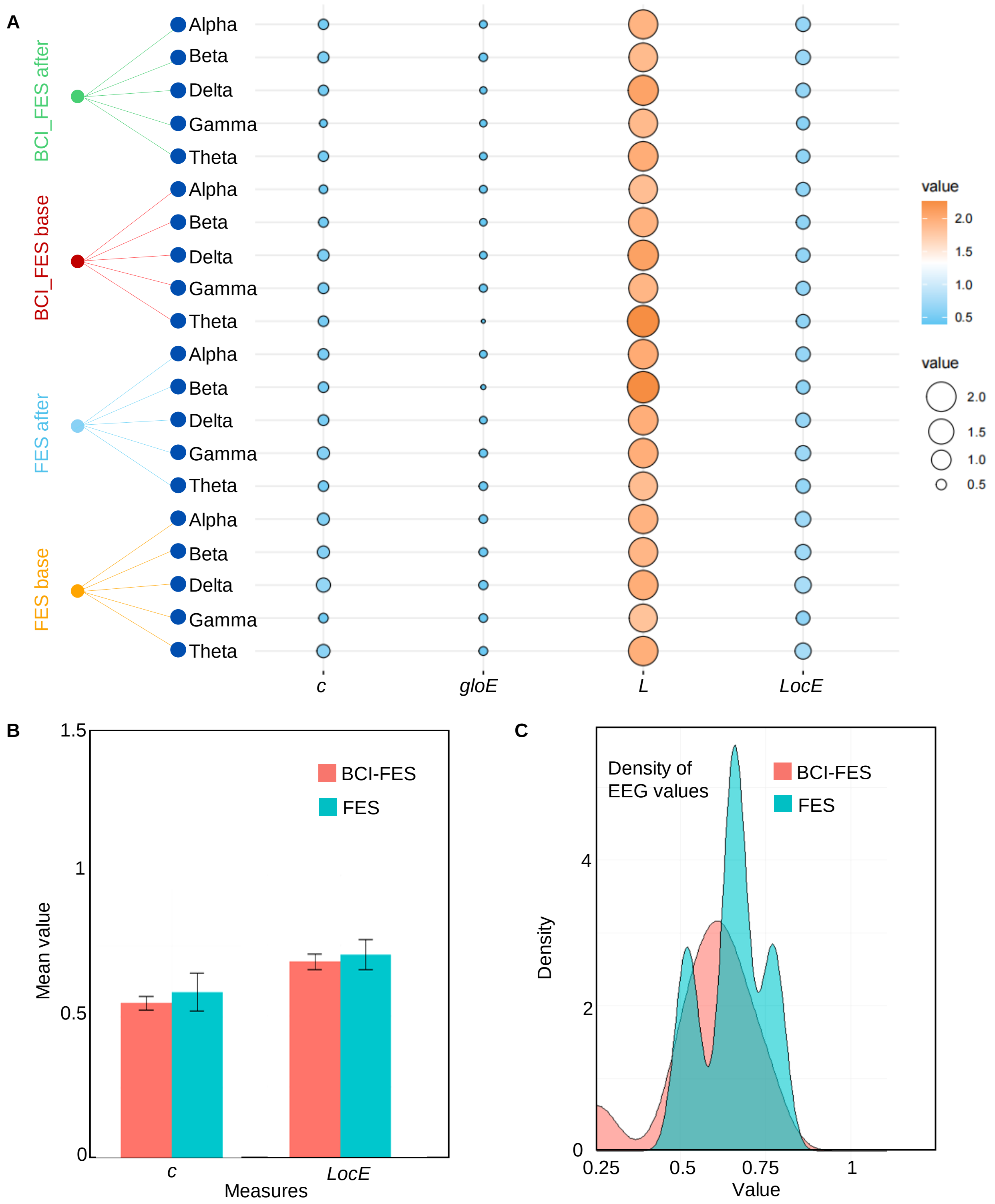

Supplementary Figure 6. EEG band power measurements across BCI-FES and FES interventions. (**A**) Visualization of EEG band power values (alpha, beta, gamma, delta, theta) for BCI-FES and FES interventions across different measures (*c*, *gLoE*, *L*, *LocE*) at baseline and after treatment. Each data point is represented by the size of the circle corresponding to the value, highlighting the differences in band power across conditions. (**B**) Bar graph representing mean values of EEG band power measures for BCI-FES (in pink) and FES (in blue), indicating the significant differences between treatment outcomes. (**C**) Density plot illustrating the distribution of EEG values for both BCI-FES and FES groups, providing insight into the variability of band power across interventions. *c* (cortical activation), *gLoE* (global localization of excitation), *L* (lateralization), and *LocE* (localization of excitation) represent cognitive and motor function assessments.

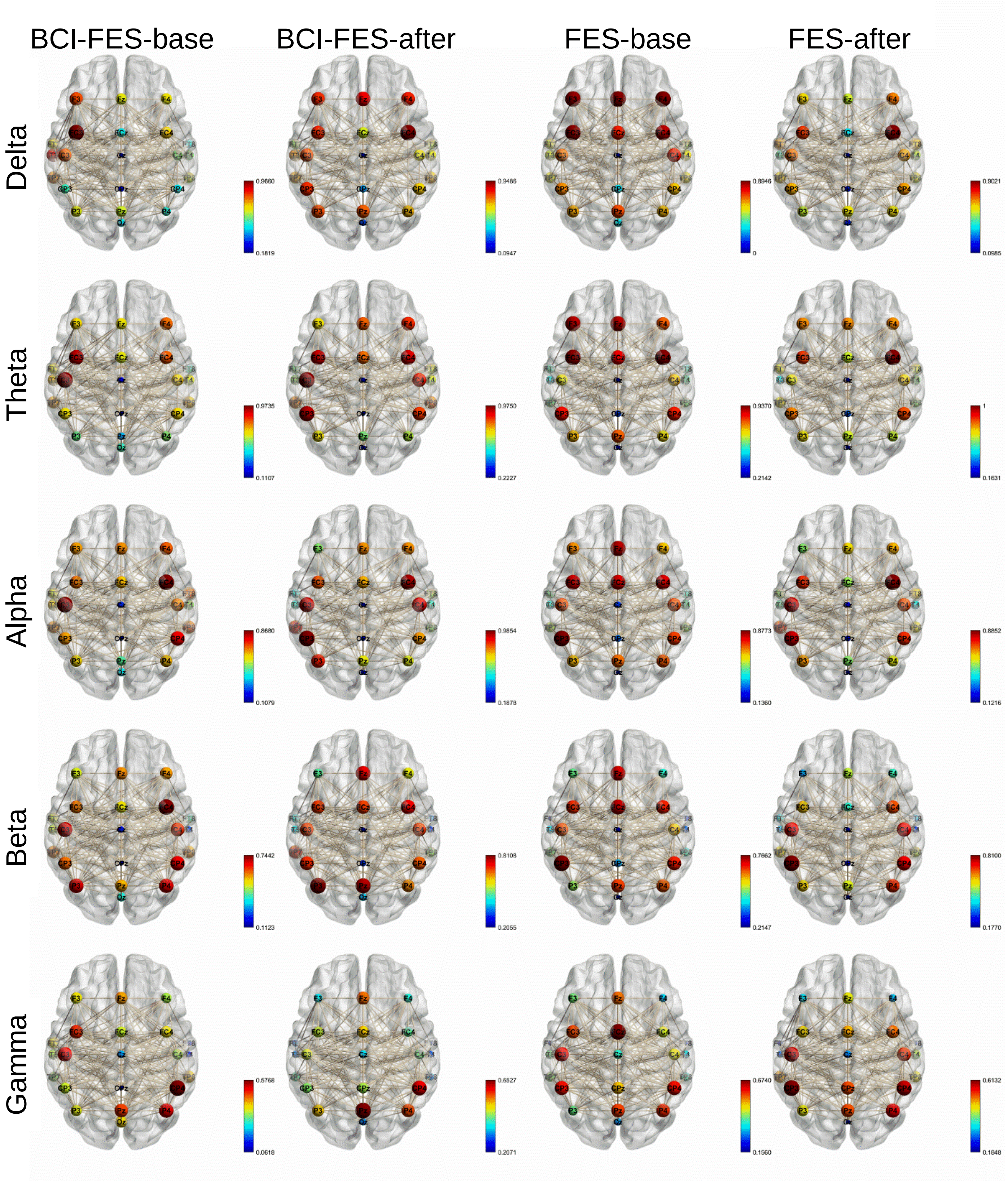

Supplementary Figure 7. Functional brain connectivity. Functional brain connectivity in the BCI-FES group and the FES group before (base) and after (aft) intervention in the delta (0.5-4 Hz), theta (4-8 Hz), alpha (8-13 Hz), beta (13-30 Hz) and gamma (30-60 Hz) bands.

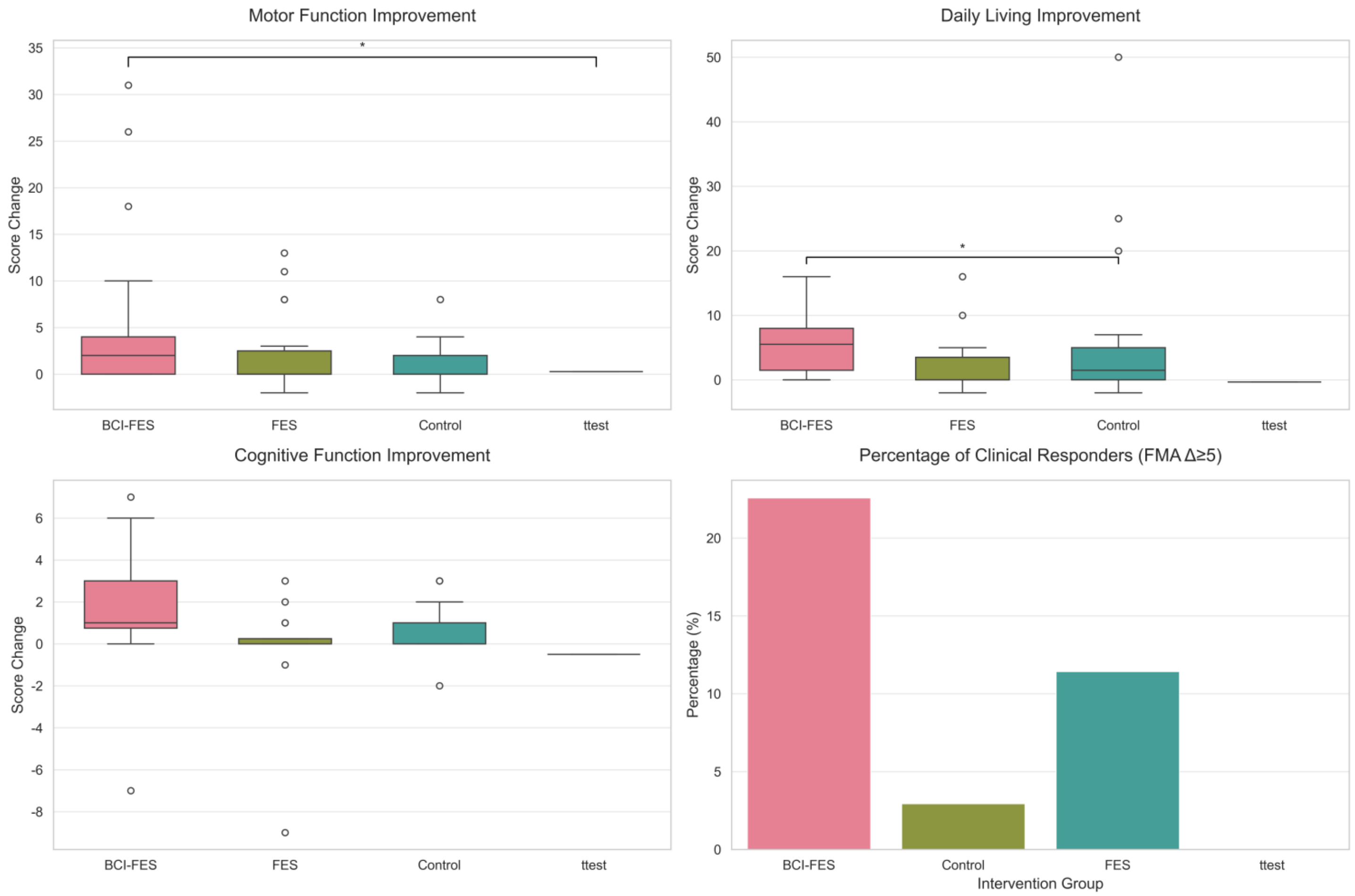

Supplementary Figure 8. Comparison of functional improvements and clinical response rates across intervention groups. BCI-FES consistently outperformed FES and Control in motor, daily living, and cognitive function improvements, and exhibited the highest rate of clinical responders.

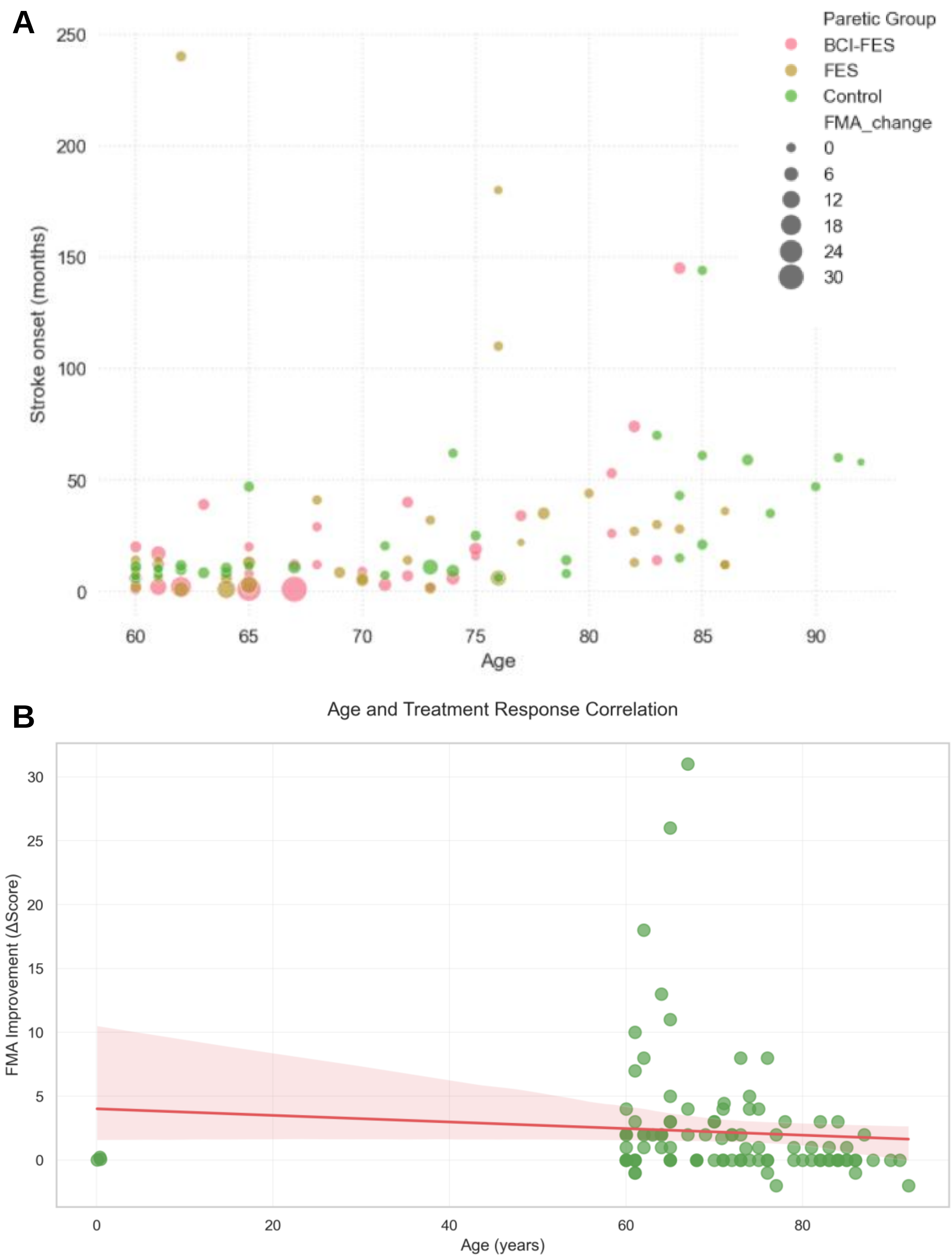

Supplementary Figure 9. Age distribution and its correlation with treatment response in stroke patients. (A) Scatter plot showing the distribution of stroke onset time (months) across different age groups (60–90 years) for three patient cohorts: BGI-FES, FES, and Control. Most patients across all cohorts exhibit stroke onset times <50 months, with no clear age-related clustering in onset duration. **(B)** Correlation between age (years) and FMA improvement score (primary treatment response metric). A weak positive trend (red line) suggests minimal association between age and FMA improvement, with the majority of patients (green points) showing FMA improvements ≤15, regardless of age. The shaded area indicates the 95% confidence interval, confirming no significant age-dependent treatment response.

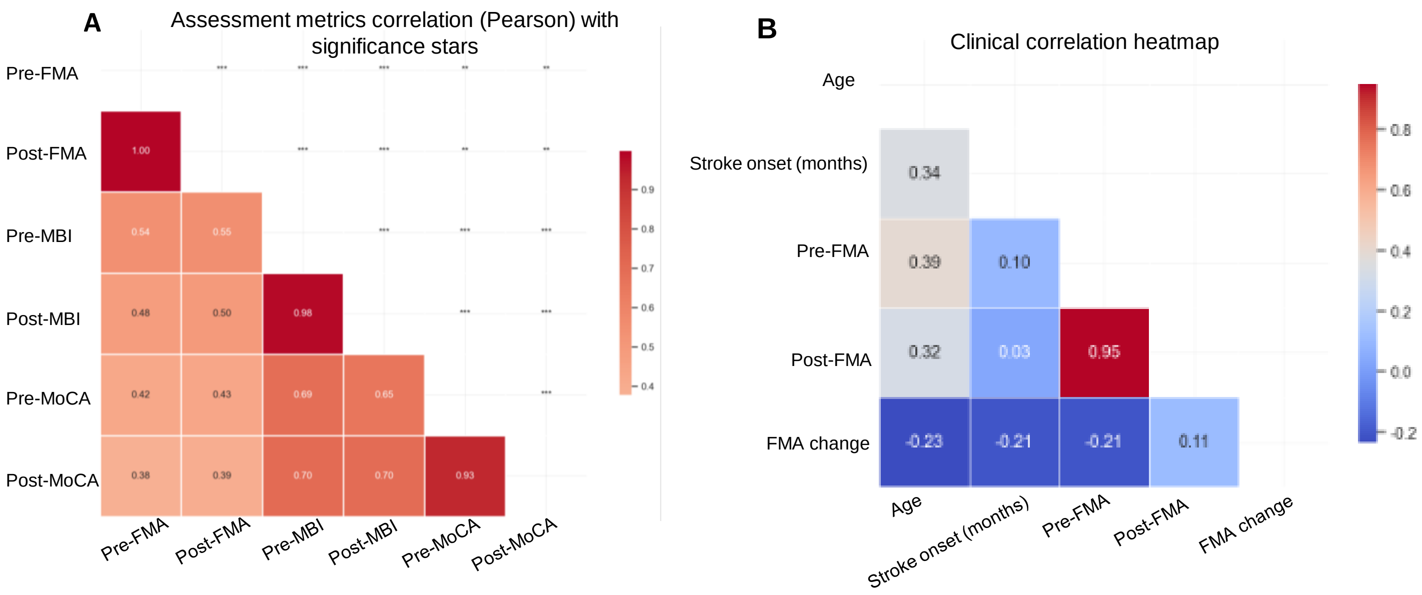

Supplementary Figure 10. Correlation analyses of assessment metrics and clinical variables. (A) Assessment metrics correlation (Pearson) with significance stars. Pearson correlation coefficients between pre- and post-intervention assessment metrics include FMA, MBI, and MCA. Strong positive correlations were observed within metrics (e.g., pre-FMA vs. post-FMA: *r*=0.96), while cross-metric correlations ranged from moderate to strong (e.g., pre-MBI vs. post-MCA: *r*=0.70). Significance stars indicate statistically significant correlations. (**B)** Clinical correlation heatmap. This heatmap illustrates Pearson correlations between clinical variables (age, stroke onset) and FMA metrics (pre-FMA, post-FMA, FMA change). Weak positive correlations were found between age and pre-FMA (*r*=0.39) and age and stroke onset (*r*=0.34). FMA change showed weak negative correlations with age (*r*=-0.23) and stroke onset (*r*=-0.21), while minimal associations were observed between FMA change and pre/post-FMA scores (*r*≤0.11).

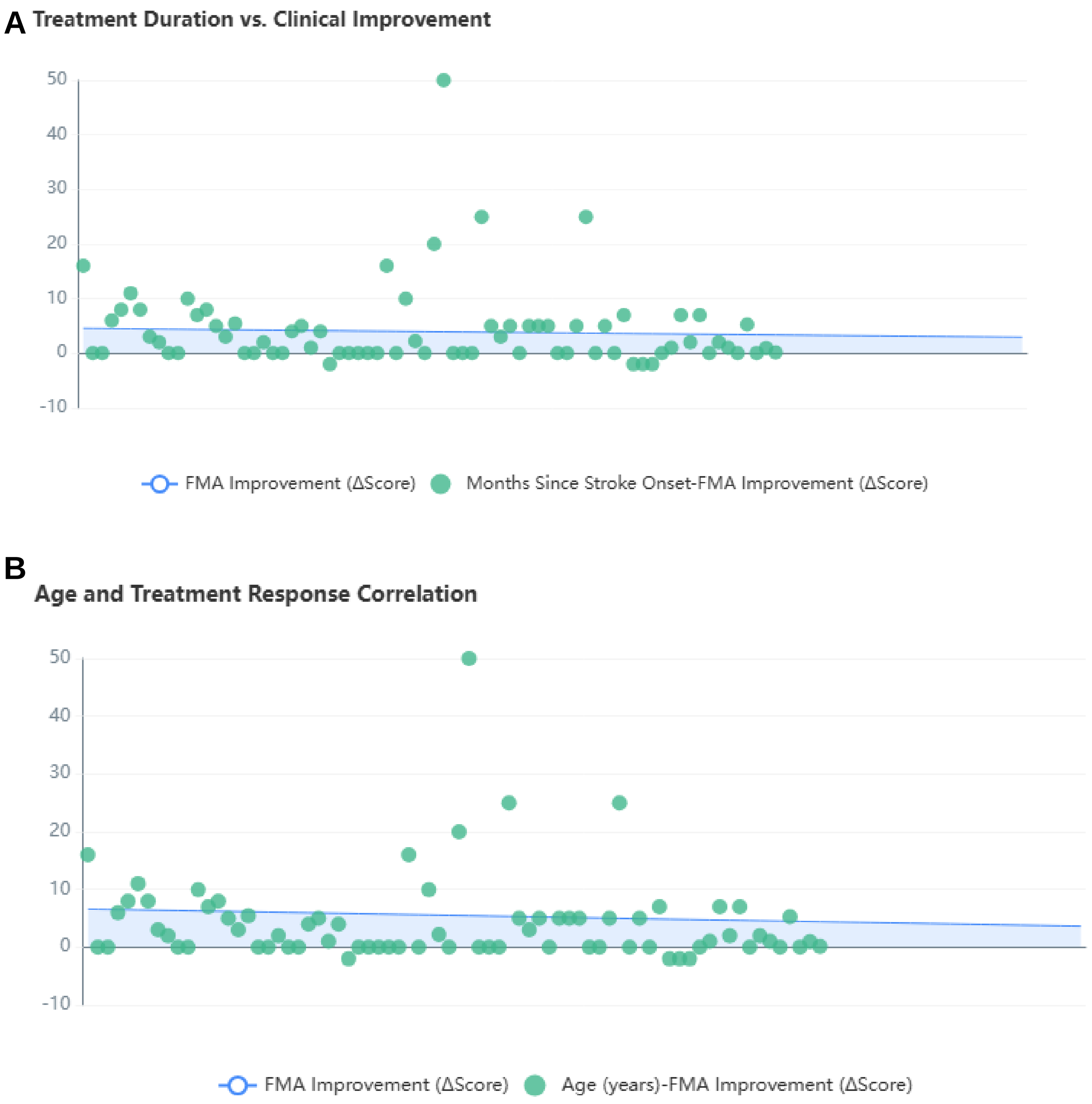

Supplementary Figure 11. Comparison of clinical improvement correlations with treatment duration and age. (**A**) Treatment duration vs. clinical improvement; Scatter plot showing the relationship between months since stroke onset and FMA improvement (ΔScore). Most data points cluster near minimal improvement (ΔScore ~0), with a few outliers showing modest positive improvement. (**B**) Age and treatment response correlation; Scatter plot depicting the association between patient age and FMA improvement, with no clear trend of age-related differences in improvement.

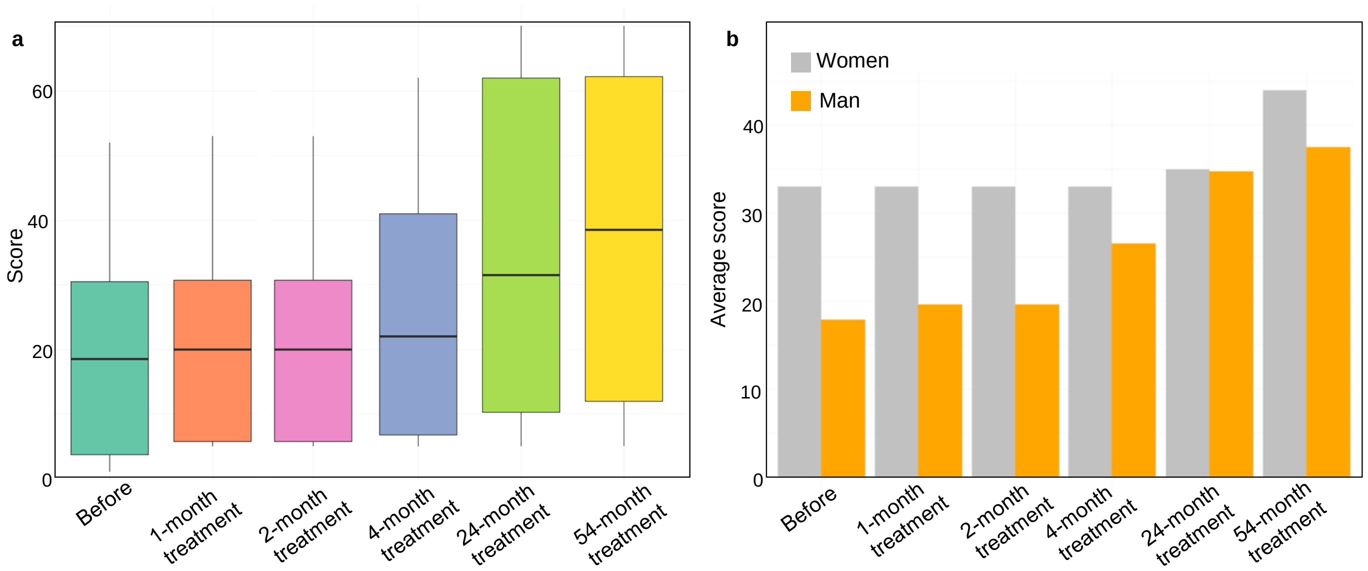

Supplementary Figure 12. Changes in treatment scores over time. (**A**) Boxplots illustrating the distribution of treatment scores before and at multiple time points during treatment (1-month, 2-month, 4-month, 24-month, and 54-month) for participants. Scores demonstrate progressive improvement across treatment phases, indicating the efficacy of the intervention. (**B**) Bar graph displaying the average treatment scores segmented by gender (women in gray and men in orange) across the same time points, revealing differences in response to treatment between participants with different genders.

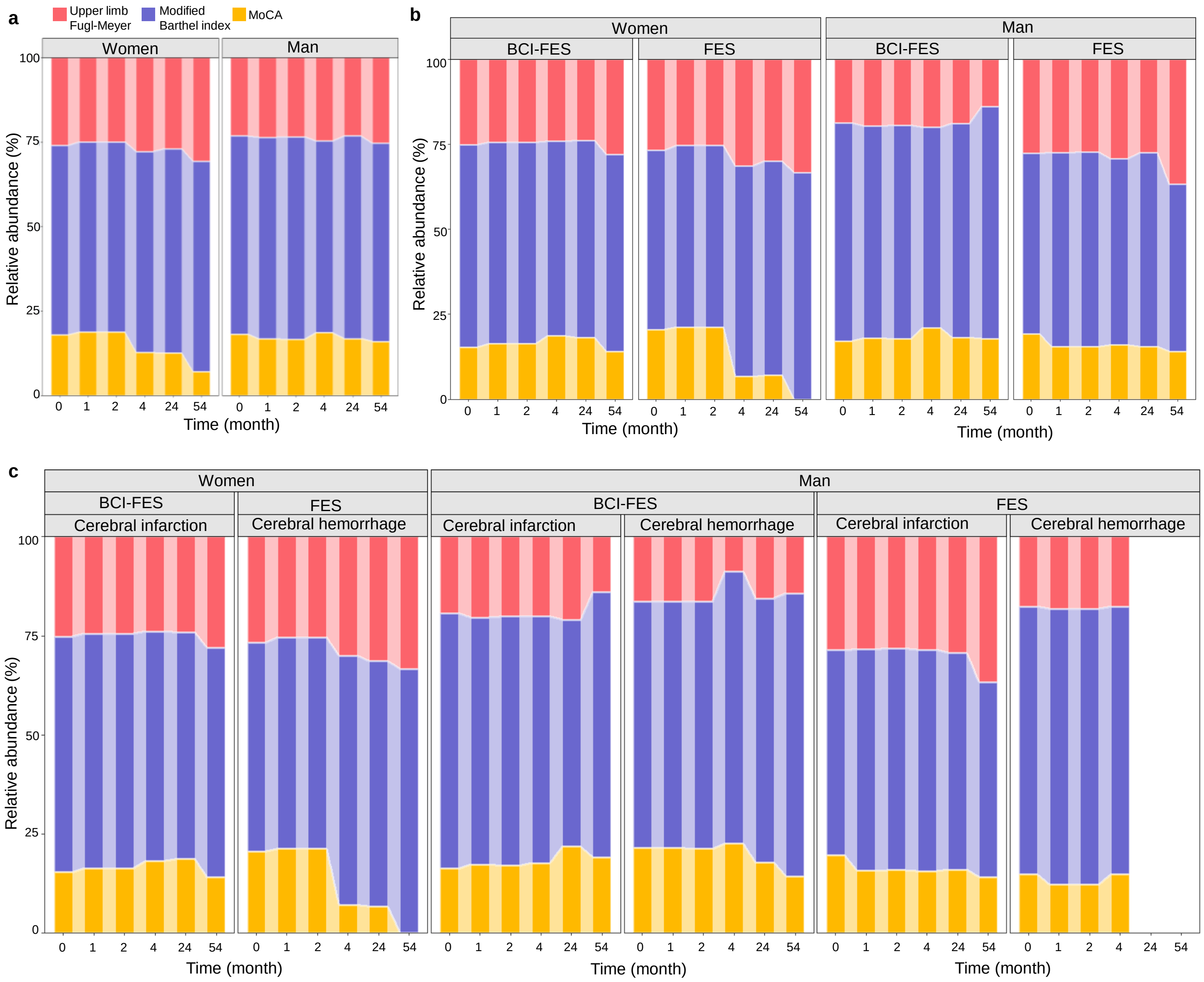

Supplementary Figure 13. Recovery assessment across time for different demographics and stroke types. (**A**) Relative abundance of recovery metrics in women and men, using scales for upper limb function (Fugl-Meyer), MBI, and MoCA over various time points (0, 1, 2, 4, 24, 54 months). (**B**) Changes in recovery metrics as stratified by BCI-FES and FES treatments for both women and men, illustrating the proportion of upper limb function, MBI, and MoCA contributions across time. (**C**) Recovery assessment based on stroke subtype (cerebral infarction vs. cerebral hemorrhage) across BCI-FES and FES treatments in different genders, showing relative abundances of recovery metrics over time. Each segment's color represents a specific assessment scale, indicating shifts in recovery dynamics over the months.
